# Effectiveness of psychological interventions in prison to reduce recidivism: a systematic review and meta-analysis of randomised controlled trials

**DOI:** 10.1016/S2215-0366(21)00170-X

**Published:** 2021-09

**Authors:** Gabrielle Beaudry, Rongqin Yu, Amanda E Perry, Seena Fazel

**Affiliations:** a Department of Psychiatry, University of Oxford, Oxford, UK; b Department of Health Sciences, University of York, York, UK

## Abstract

**Background:**

Repeat offending, also known as criminal recidivism, in people released from prison has remained high over many decades. To address this, psychological treatments have been increasingly used in criminal justice settings; however, there is little evidence about their effectiveness. We aimed to evaluate the effectiveness of interventions in prison to reduce recidivism after release.

**Methods:**

For this systematic review and meta-analysis, we searched the Cochrane Central Register of Controlled Trials, Embase, Global Health, MEDLINE, PsycINFO, and Google Scholar for articles published from database inception to Feb 17, 2021, without any language restrictions. We searched for randomised controlled trials (RCTs) that evaluated the effect of psychological interventions, delivered to adolescents and adults during incarceration, on recidivism outcomes after release. We excluded studies of solely pharmacological interventions and of participants in secure psychiatric hospitals or special residential units, or attending therapies mainly delivered outside of the prison setting. We extracted summary estimates from eligible RCTs. Data were extracted and appraised according to a prespecified protocol, with effect sizes converted to odds ratios. We used a standardised form to extract the effects of interventions on recidivism and estimated risk of bias for each RCT. Planned sensitivity analyses were done by removing studies with fewer than 50 participants. Our primary outcome was recidivism. Data from individual RCTs were combined in a random-effects meta-analysis as pooled odds ratios (ORs) and we explored sources of heterogeneity by comparing effect sizes by study size, control group, and intervention type. The protocol was pre-registered with PROSPERO, CRD42020167228.

**Findings:**

Of 6345 articles retrieved, 29 RCTs (9443 participants, 1104 [11·7%] females, 8111 [85·9%] males, and 228 [2·4%] unknown) met the inclusion criteria for the primary outcome. Mean ages were 31·4 years (SD 4·9, range 24·5–41·5) for adult participants and 17·5 years (SD 1·9; range 14·6–20·2) for adolescent participants. Race or ethnicity data were not sufficiently reported to be aggregated. If including all 29 RCTs, psychological interventions were associated with reduced reoffending outcomes (OR 0·72, 95% CI 0·56–0·92). However, after excluding smaller studies (<50 participants in the intervention group), there was no significant reduction in recidivism (OR 0.87, 95% CI 0·68–1·11). Based on two studies, therapeutic communities were associated with decreased rates of recidivism (OR 0·64, 95% CI 0·46–0·91). These risk estimates did not significantly differ by type of control group and other study characteristics.

**Interpretation:**

Widely implemented psychological interventions for people in prison to reduce offending after release need improvement. Publication bias and small-study effects appear to have overestimated the reported modest effects of such interventions, which were no longer present when only larger studies were included in analyses. Findings suggest that therapeutic communities and interventions that ensure continuity of care in community settings should be prioritised for future research. Developing new treatments should focus on addressing modifiable risk factors for reoffending.

**Funding:**

Wellcome Trust, Fonds de recherche du Québec – Santé.

## Introduction

11 million people are currently held in jails or prisons worldwide and every year 30 million individuals enter and leave custody.^1^, ^2^ People released from jails or prisons have a higher risk of repeat offending than people given community-based sanctions, and account for nearly a fifth of all new crimes annually.^3^ Typically, between a third and a half of people released from prison reoffend within 2 years.^4^ The societal costs of recidivism are considerable, and include public health and associated economic effects. For example, the annual social and economic cost of reoffending is estimated at more than £18·1 billion in the UK and US$13 billion in one US large state (Illinois) alone.^5^, ^6^

Various psychological interventions have been used in custodial settings to improve outcomes for people released from prison, and to reduce reoffending in particular. Some reviews suggested that cognitive behavioural therapy (CBT) programmes are among the most effective interventions, with meta-analyses reporting recidivism risk reductions of 20–30%.^7^, ^8^, ^9^, ^10^, ^11^, ^12^, ^13^ Furthermore, treatment programme adherence to risk–need–responsivity principles^14^ is associated with reductions in reoffending; however, this link is based on predominantly quasi-experimental studies.^15^, ^16^, ^17^ Overall, the effectiveness of most prison-based treatments on recidivism remains unclear because the evidence is inconsistent and subject to a range of limitations.^18^, ^19^, ^20^, ^21^, ^22^, ^23^, ^24^, ^25^, ^26^, ^27^, ^28^ Previous reviews have often focused on specific groups—eg, women,^26^, ^29^ adolescents,^20^, ^23^ individuals who use drugs,^25^ people living with a mental health condition,^18^ and people with sexual^21^, ^28^ or other violent^19^, ^27^ index offences. There are considerable methodological differences between these reviews, particularly in the quality of included primary studies,^20^ and the sources of this heterogeneity have rarely been examined.^18^ Also, existing reviews have pooled estimates that combine samples from diverse settings (eg, prisons and secure psychiatric hospitals)^24^ or were published before 2008.^19^, ^22^, ^23^, ^29^ To address these limitations, we aimed to synthesise reoffending outcomes from all randomised controlled trials (RCTs) of psychological interventions provided in prisons.

**Research in context**

**Evidence before this study**

We searched the Cochrane Central Register of Controlled Trials, EMBASE, Global Health, MEDLINE, PsycINFO from database inception to Feb 17, 2021, for systematic reviews and meta-analyses of the effectiveness of psychological interventions delivered in prisons, without language restrictions. We used similar keywords across databases relating to psychological interventions (eg, program*, intervention*, treatment*), incarceration (eg, prison*, incarcerat*, custod*), and recidivism (eg, recommit*, reoffend*, recidiv*). We identified several relevant systematic reviews, but none provided a comprehensive overview of the evidence base, as their scope was limited to specific groups of individuals (eg, people with co-occurring mental illness or people in specific offence categories), or certain types of intervention (eg, CBT). Furthermore, previous reviews have included studies using non-experimental designs, which are liable to overestimate effects. Despite this limitation, these reviews stated that some psychological interventions (eg, CBT and risk–need–responsivity therapies) are effective in reducing recidivism on release from prison.

**Added value of this study**

We did a comprehensive systematic review and meta-analysis of all randomised controlled trials that evaluated the effectiveness of psychological interventions delivered in prisons on recidivism outcomes after release. We provide an up to date systematic review, which is both broader in scope (by including all prisoners irrespective of criminal history, setting, or psychological treatment) and more precise (by including only randomised controlled trials) than previous reviews. The effects were considerably smaller than expert opinion had previously maintained, with no clear effects of CBT-based treatments.

**Implications of all the available evidence**

Psychological treatments, which were developed to treat mental health conditions, need to be adapted to target modifiable risk factors that are specific to reoffending. Continued treatment after prison release should be integrated into therapeutic programmes. The evidence is inconclusive for most psychological interventions, and the findings of this systematic review could inform how different treatment modalities should be prioritised in service development and future trials.

## Methods

### Search strategy and selection criteria

For this systematic review and meta-analysis, we searched Cochrane Central Register of Controlled Trials, Embase, Global Health, MEDLINE, PsycINFO, and Google Scholar for RCTs published from database inception until Feb 17, 2021. The search strategy combined terms relating to RCTs (ie, random*, trial*, placebo*), psychological interventions (eg, program*, intervention*, treatment*), incarceration (eg, prison*, incarcerat*, custod*), and recidivism (eg, recommit*, reoffend*, recidiv*). For the full list of search terms see appendix pp 3–7. We also manually searched the reference lists of included studies, and relevant articles and systematic reviews.

We included RCTs of psychological interventions in jails and prisons that reported on criminal recidivism occurring after release from prison as an outcome. Studies were eligible for inclusion if they met the following criteria: RCT (including pilot studies and cluster-randomised trials); all participants were incarcerated at the time of random allocation (including adolescents, people in custody awaiting trial, and people residing in immigration detention centres) and remained incarcerated for the duration of the treatment; participants assigned to control groups were exposed to the usual intervention, no intervention, or an alternative intervention to the experimental group; intervention was psychological (eg, CBT or mindfulness-based therapy) or psychoeducational (eg, vocational or educational training); interventions (both individual and group formats) were delivered in a jail or prison setting; and the recidivism outcome (eg, reconviction, reincarceration, rearrest, parole violation, or new charges) was reported separately for the intervention and control groups. We included studies in which post-prison services were offered to participants on a voluntary basis, but were not directly part of the evaluated intervention (eg, the Challenge to Change,^30^ and the Amity therapeutic community programmes^31^). We excluded studies on the basis of the following criteria: trial not randomised (eg, case studies and pretest–post-test comparisons); participants were not in jail or prison at the time of the study (eg, they were on parole, in a secure psychiatric hospital, attending therapies outside of the prison setting, or residing in community-based special residential units formerly known as bootcamps); the control group included primarily people who dropped out or refused treatment altogether; the intervention was based solely on a pharmacological approach; and the study compared jail or prison with a community sanction (eg, prison *vs* bootcamp) or involved a joint prison and community programme for which the community component accounted for more than half of the intervention duration (eg, the CREST programme^32^, ^33^). There was no limit on the follow-up time period for reoffending. Non-English language studies were translated and considered for inclusion.

One author (GB) did the searches and screened the titles and abstracts of the studies identified using the search strategy and screened the full text of those matching the predetermined inclusion criteria. In cases of uncertainty, GB consulted with RY and consensus was reached about study selection. SF resolved any disagreements about inclusion and verified the eligibility of included studies. GB extracted summary estimates from eligible RCTs.

This systematic review was done in accordance with the Preferred Items for Systematic Reviews and Meta-Analyses guidelines^34^ (appendix pp 1–2).

### Data analysis

We extracted from eligible studies information for: year of publication; geographical location; correctional setting; sample size; sex; ethnicity (Asian, Black or African American, White, Hispanic or Latinx, Indigenous, and Other); average age of participants; follow-up period for recidivism; intervention length, type, and format; definition of recidivism; and numbers of individuals in the intervention and control groups by recidivism status (ie, having reoffended *vs* not having reoffended). If there were multiple assessments of recidivism in a study, we used the most serious outcome for the meta-analysis (eg, reconviction was preferred to rearrest). For samples that featured both males and females but for which the recidivism outcome was not reported separately by sex, those including at least 90% males were recorded as males, whereas those with fewer than 90% males were recorded as both. If multiple articles were available for a given study (eg, the Amity therapeutic community programme^35^, ^36^), we included the article with the longest follow-up period for recidivism.^32^ We contacted relevant study authors if additional data or clarifications were required.

The quality of RCTs was assessed using the Cochrane Collaboration's risk-of-bias tool for randomised trials (RoB 2). Each RCT was given an overall estimation of risk of bias (ie, low risk, some concerns, or high risk) according to the following domains for risk of bias: randomisation process; deviations from intended interventions; missing outcome data; measurement of the outcome; and selection of the reported result.^37^ Trials with a high risk of bias in at least one domain were rated as having a high risk of bias.

The primary outcome was recidivism. This measure was assessed with the summary odds ratio (OR) and corresponding 95% CI. We sought both continuous and dichotomous data on recidivism. To enable comparison across studies, when the outcome was given as continuous data, we first attempted to obtain the equivalent dichotomous data from the authors of the primary studies. If we were unable to do so, we converted the standardised mean difference to ORs (using the formula recommended by the Cochrane Handbook^38^). One study was excluded because of insufficient information.^39^ Furthermore, for multiarm trials,^40^, ^41^ two distinct approaches recommended by the Cochrane Handbook^38^ were used to avoid double-counting participants in the shared control group. For one study,^40^ we merged both intervention arms into a single comparison group, as they both were psychoeducational interventions. For another study,^41^ we included each pairwise comparison separately (one was psychoeducational and the other CBT-based) by evenly dividing the shared control group among the comparisons.

We did a random-effects meta-analysis to estimate the effect sizes, because this gives similar weights to studies with different sample sizes and substantial heterogeneity was expected between studies (eg, for type and length of interventions and follow-up periods). Pooled OR estimates were grouped into domains and summarised using forest plots. Between-study heterogeneity was estimated using Cochran's *Q* (reported with a χ^2^-value and p value) and the *I*^2^ statistic. Amounts of heterogeneity were evaluated according to thresholds: low (0–40%), moderate (30–60%), substantial (50–90%), and considerable (75–100%).^38^ These heterogeneity measures should be interpreted with caution if the number of studies is small (eg, in subgroup analyses).^42^

We first pooled all individual RCTs to calculate the summary effect size. We then stratified studies according to whether the psychological intervention group was larger than 50 participants.^31^, ^32^, ^43^, ^44^, ^45^, ^46^, ^47^, ^48^, ^49^, ^50^, ^51^, ^52^, ^53^, ^54^, ^55^, ^56^ This cutoff was determined in accordance with previous research on randomised experiments (eg, psychotherapy for adult depression^57^) to maximise the key beneficial effect of randomisation (ie, controlling for unknown and unmeasurable variables^58^, ^59^), and rule out potential small- study effects.^60^ Among these studies, we explored the effects of control group (ie, usual care, wait-list, and other) and intervention type (ie, CBT-based, psychoeducational, therapeutic communities, and other), and excluded two studies^43^, ^56^ from the secondary analysis on the basis of considerable differences in treatment duration (eg, one session only)^56^ and delivery mode (eg, video feedback of previous sessions^43^). All interventions based on cognitive behavioural approaches were considered to be CBT-based psychological interventions.^44^, ^45^, ^46^, ^47^, ^49^, ^55^ Interventions with a core vocational or educational component (eg, deterrence^51^) were included in the psychoeducational category.^50^ Interventions of therapeutic communities formed another category.^30^, ^31^ Both therapeutic community trials included voluntary post-prison services. Most (83%) participants from the Challenge to Change trial^30^ chose to access community-based mental health or substance abuse services, although these were beyond the scope of that study. The Amity therapeutic community offered residential treatment to programme graduates (experimental group only) at an Amity-operated facility called Vista.^31^ The effect of Vista on recidivism was not considered in our meta-analysis, to avoid annulling the effects of randomisation; however, we reported percentages in the Discussion. The other intervention category combined reality therapy,^48^ social therapy,^5^ interactive journaling,^54^ and gender-responsive substance abuse therapy.^52^

Prespecified subgroup (mixed-effects) and meta-regression analyses were done to examine sources of heterogeneity. The following study characteristics were assessed: year of publication (<1990 *vs* ≥1990; to account for the formalisation of the risk-need-responsivity model in 1990),^14^ study location (USA *vs* elsewhere), sample size (as a continuous variable), sex (sex-specific interventions *vs* those delivered to both males and females simul-taneously), mean participant age (as a continuous variable), age group (adolescents *vs* adults), intervention type (CBT-based *vs* all other types), comparator type (usual care *vs* waitlist or other), follow-up time period (as a continuous variable), intervention format (individual *vs* group or combination), intervention aimed at substance use disorder (as a dichotomous variable) and risk of bias (high *vs* low or some concerns).

We did influence analysis on all studies to determine which of them disproportionately influenced the summary effect of our meta-analysis. We used the leave-one-out method and showed results using the Baujat plot.^61^

We examined publication bias in all studies using the Egger's test of the intercept^62^ and funnel plot analysis. If the Egger's test reported publication bias and between-study heterogeneity was not substantial,^63^ we followed the trim and fill procedure^64^ to correct for publication bias by imputing missing studies into a new symmetrical funnel plot.^65^

If the results of the publication bias analysis indicated small-study effects, we did further sensitivity analyses. First, we compared the fixed-effect and random-effect estimates of the intervention effect, because a more favourable estimate in the random-effects model might indicate that interventions were more effective in smaller studies. We did an additional analysis by only including studies with an intervention group of at least 100 participants.^30^, ^31^, ^44^, ^46^, ^47^, ^49^, ^50^, ^53^, ^55^ We did this to reduce small-study effects, and to evaluate the robustness of the findings, as small trials are susceptible to selection bias and tend to have larger treatment effects than large trials.^65^, ^66^ We also investigated the effect of study quality on the pooled effect size, by removing studies at high risk of bias.

All statistical analyses were done in R version 3.6.2 and R Studio version 1.4.1717.^67^, ^68^ The study protocol was registered with PROSPERO, CRD42020167228.

### Role of the funding source

The funders of the study had no role in study design, data collection, data analysis, data interpretation, or writing of the report.

## Results

**Figure 1:**
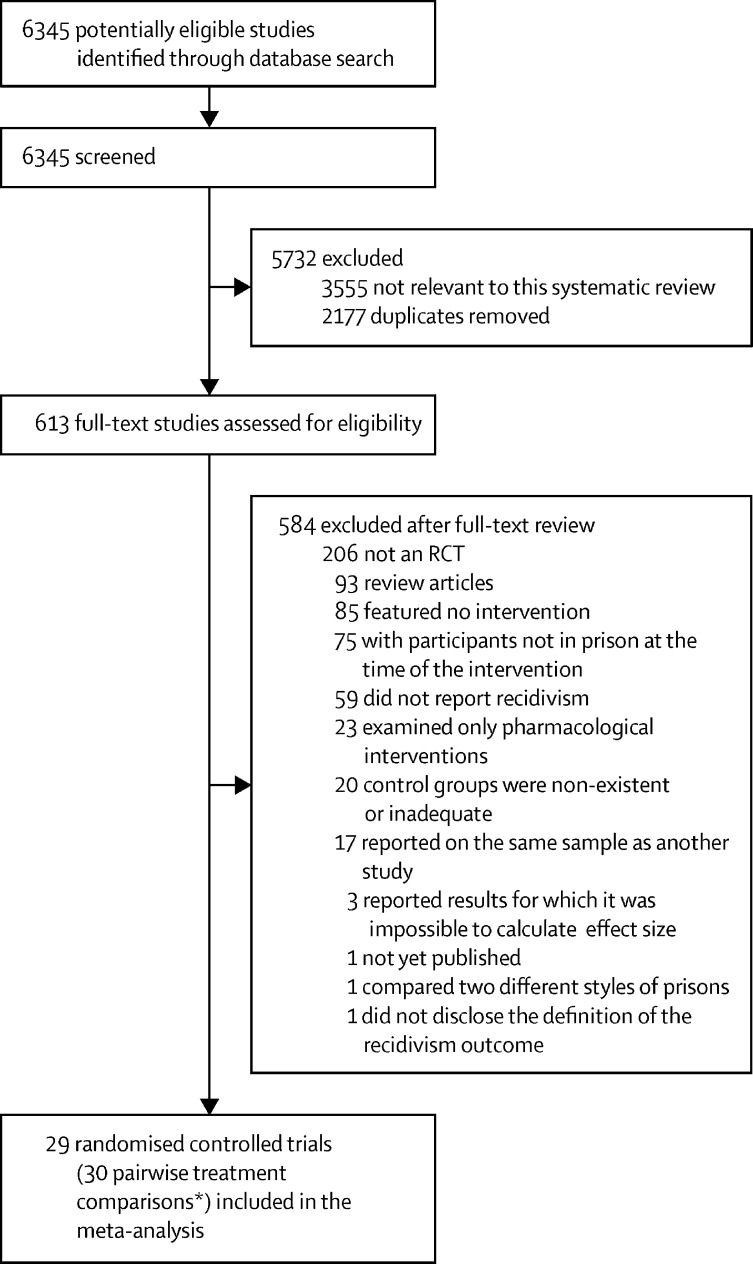
Study selection *The 29 randomised controlled trials, included 27 RCTs that were two-arm trials and two that were three-arm trials.^40^, ^41^ Overall, the trials described 31 psychological interventions that were combined into 30 pairwise treatment comparisons on which the statistical analyses were based.

We identified 6345 articles through electronic searches and 29 eligible trials (for selection process see figure 1 and for study characteristics see table 1).^30^, ^31^, ^40^, ^41^, ^43^, ^44^, ^45^, ^46^, ^47^, ^48^, ^49^, ^50^, ^51^, ^52^, ^53^, ^54^, ^55^, ^56^, ^69^, ^70^, ^71^, ^72^, ^73^, ^74^, ^75^, ^77^, ^78^, ^79^, ^80^ Most RCTs were two-arm trials (n=27); two were three-arm trials.^40^, ^41^ These trials described 31 psychological interventions that were combined into 30 pairwise treatment comparisons, on which the statistical analyses were based. In total, 9443 individuals (1104 [11·7%] females, 8111 [85·9%] males, and 228 [2·4%] individuals for whom sex was not reported) participated in the trials, and 6528 (1118 [17·1%] adolescents and 5410 [82·9%] adults) had recidivism outcome data. The mean age was 31·4 years (SD 4·9, range 24·5–41·5) in adults and 17·5 years (1·9, 14·6–20·2) in adolescents. Descriptive statistics on the age of participants were calculated using the mean age from each study and the range of mean ages (if available). Race or ethnicity data from each study are summarised in the appendix (pp 8–9). Among included trials, 19 were from the USA (n=3578 [54·8%]),^30^, ^31^, ^41^, ^44^, ^46^, ^47^, ^48^, ^49^, ^50^, ^51^, ^52^, ^54^, ^69^, ^71^, ^72^, ^74^, ^75^, ^78^, ^79^ four from Canada (n=2351),^40^, ^43^, ^55^, ^70^ two from the UK (n=203);^45^, ^56^ and one each from Germany (n=223),^53^ Sweden (n=59),^73^ Japan (n=50),^77^ and Norway (n=64).^80^ Treatment duration varied considerably between trials, ranging from one session only^56^ to multiple interventions that lasted for 1 year.^31^, ^74^ The most frequent source of trial funding was government-funded research council. None of the psychological interventions was described as being mandatory and recruitment of participants was voluntary. However, it is possible that perceived coercion and other incentives could have contributed to the decision to participate.

**Table 1:** Characteristics of randomised controlled trials of psychological interventions in prison to reduce recidivism

	**Country**	**Setting**	**Participants randomly allocated**	**Participants followed up (%)**	**Sex**	**Mean age, years (SD)**	**Psychological intervention; category; format**	**Comparator**	**Duration of intervention and number or frequency of sessions**	**Detailed definition of recidivism outcome**	**Follow-up period of recidivism**
Persons (1967)^69^	USA	Institution for boys	82	82 (100%)	Males	16·4 years (SD not reported)	Psychotherapy; other; combination	No treatment	20 weeks (80 h over 60 sessions) total; twice per week group psychotherapy (1·5 h per session) plus an average of 1 h per week individual psychotherapy	Reinstitutionalisation in any penal institution	Mean 9·5 months (further details not reported)
Annis (1979)^43^	Canada	Minimum-security institution	150	128 (85%)	Males	24·5 years (range 18–64; SD not reported)	Awareness group (with and without video feedback); psychoeducational; group	Routine institutional care	8 weeks total; mean 224 h of programme sessions (further details not reported)	Incarcerated at follow-up	1 year
Lewis (1983)^51^	USA	Four camps	108	108 (100%)	Males	16·3 years (range 14–18; SD not reported)	Squires programme; psychoeducational; group	No treatment	3 consecutive Saturday morning sessions (3 h per session)	Subsequent arrest, or charge, or both	1 year
Linden et al (1984)^70^	Canada	Two penitentiaries (maximum and medium security)	66	55 (83%)	Males	Not reported	Prison educational programme; psychoeducational; combination	No treatment	Not reported	Marginal failure (ie, return to prison for minor crime or technical violation of parole regulations) or clear recidivism (ie, return to prison for major offence)	77–82 months
Homant (1986)^71^	USA	Prison	92	86 (93%)	Males	Not reported	Group therapy; other; group	Standard care (control group participants were free to seek out therapy [group or individual] through the usual channels)	Mean number of therapy sessions during the first year of imprisonment: 18·6 experimental group, 4·0 control group (further details not reported)	Reincarceration for a new felony (ie, serious criminal offence) or reincarceration on felony (ie, breach of post-release supervision conditions)	10 years
Shivrattan (1988)^40^	Canada	Institution for incarcerated delinquents	45	42 (93%)	Males	Mean not reported (range 15–17 years)	Social interaction skills programme and stress management training programme; psychoeducational; group	No treatment	8 sessions (further details not reported)	Further criminal activity (ie, being charged and sentenced to incarceration in an institution)	12–15 months
Guerra and Slaby (1990)^41^	USA	Juvenile correctional facility	165	83 (50%)	Both (50% females, 50% males)	17·2 years (range 15–18; SD not reported)	Cognition mediation training plus attention control; CBT-based and psychoeducational; group	No treatment	12 weeks total; once a week meetings (1 h per session)	Parole violation	≥1 year and ≤2 years
Lattimore et al (1990)^50^	USA	Prison	591	247 (42%)	Males	20·0 years (SD not reported)	Vocation delivery system; psychoeducational; group	Routine care (eg, assignment to the first available vocational training programme or to a prison job)	Not reported	Rearrest	Mean 2 years (range 411–1530 days; further details not reported)
Leeman et al (1993)^72^	USA	Medium-security correctional facility	57	57 (100%)	Males	16·0 years (range 15–18 years; SD not reported)	Equipping youth to help one another; CBT-based; group	Simple or motivational therapy	1–1·5 h, 5 days per week	Parole revocation, or recommitted to an institution, or both	6 and 12 months
Robinson (1995)^55^	Canada	Correctional facility	4072	2125 (52%)	Males	29·6 years (SD 7·2)	Cognitive skills training; CBT-based; group	Waitlist	36 sessions	Reconviction for a new offence	1 year
Lindforss and Magnusson (1997)^73^	Sweden	Prison	60	59 (98%)	Males	Not reported	Solution-focused brief therapy; other; individual	No treatment	Not reported	Committed further offence that resulted in a sentence to probation or imprisonment	12 and 16 months
Dugan and Everett (1998)^48^	USA	Jail	145	117 (81%)	Males	30·2 years (SD 9·0)	Reality therapy; other; group	No treatment	72 h total	Mean number of offence charges	2 years
Ortmann (2000)^53^	Germany	Prison	228	223 (98%)	Not reported	Not reported	Social therapy; other; not reported	No treatment	Not reported	Any new sentences given	5 years
Armstrong (2003)^44^	USA	Young offenders unit in a detention centre	256	212 (83%)	Males	20·2 years (range 15–22; SD 1·0)	Moral reconation therapy; CBT-based; group	No treatment	1–1·5 h, on average 3 sessions per week	Arrest followed by a conviction for which time in jail or prison was levied and served	Mean 563 (median 568) days treatment group, mean 617 (median 632) days control group
Prendergast et al (2004)^31^	USA	Medium-security prison	715	576 (81%)	Males	30·7 years	Amity therapeutic community programme; therapeutic communities; group	No treatment	1 year total	Reincarceration	5 years
Sacks et al (2004)^74^	USA	Prison	236	107 (45%)	Males	34·3 years (SD 8·8)	Prison modified therapeutic community plus aftercare; therapeutic communities; group	Mental health treatment programme	1 year total	Reincarceration	1 year
Shapland et al (2008)^56^	UK	Prison	94	94 (100%)	Males	Not reported	Justice research consortium restorative justice scheme; other; individual	No treatment	One conferencing session	Reconviction	2 years
Zlotnick et al (2009)^75^	USA	Residential substance abuse treatment programme in a minimum security wing of a women's prison	49	44 (90%)	Females	34·6 years (SD 7·4)	Seeking Safety plus treatment as usual; CBT-based; group	Treatment as usual (similar to other US state prison programmes for substance users)	6–8 weeks total; 90 min sessions, typically 3 times per week	Reincarceration	6 months
Messina et al (2010)^52^	USA	Women's prison	115	115 (100%)	Females	35·9 years (SD 9·6)	Gender responsive therapy using manualised curricula (Helping Women Recover; Beyond Trauma); other; group	Standard prison therapeutic community programme	Helping Women Recover (17 sessions) and Beyond Trauma (11 sessions)	Reincarceration	1 year
Proctor et al (2012)^54^	USA	Jail	185	183 (99%)	Males	36·6 years (SD 11·1)	Interactive journalling; other; individual	Placebo (government booklet on substance misuse disorders and criminal behaviour)	Not reported	Being booked (ie, processed after arrest) in the county jail	1 year
Sacks et al (2012)^30^	USA	Women's correctional facility	468	370 (79%)	Females	35·1 years (SD 7·9)	Challenge to change therapeutic community; therapeutic communities; group	CBT-based intervention for substance misuse	Planned 6 months tenure; programme activities were provided 4 h per day, 5 days per week	Reincarceration	1 year
Bowes et al (2014)^45^	UK	Two medium-security prisons	115	109 (95%)	Males	24·5 years (SD 5·7)	Control of violence for angry, impulsive drinkers plus treatment as usual; CBT-based; group	Treatment as usual	4 weeks total; 10 sessions; approximately 20 h of group treatment and ≥4 h of individual support	Reconviction	Mean 518 days (SD 264)
Yokotani and Tamura (2015)^77^	Japan	Prison	50	50 (100%)	Males	41·5 years (SD 10·5)	Personalised feedback intervention; other; individual	No treatment	3 months; six personalised feedback letters; letter sent twice per month	Reincarceration	Mean 3·6 years (range 0·1–5·8)
Chaple et al (2016)^47^	USA	Ten prisons	494	482 (98%)	Both (31·4% females, 69·6% males)	36·6 years (SD 9·6)	Experimental condition therapeutic education system; CBT-based; individual	Standard care	12 weeks total; 48 interactive, multimedia modules; once a week for 2 h or twice per week for 1 h (depending on laboratory availability)	Reincarceration	1 year
Kubiak et al (2016)^78^	USA	Prison for women	42	35 (83%)	Females	33·7 years (SD 8·9)	Beyond violence; other; group	Treatment as usual	20 sessions; 40 h total	Reincarceration	1 year
Burraston and Eddy (2017)^46^	USA	Four US state correctional facilities (releasing institutions)	359	359 (100%)	Both (55% females, 45% males)	31·4 years (SD not reported)	Parent management training CBT-based; group	Services as usual	12 weeks total, 2·5 h sessions, three times per week	Mean number of post-release arrests	1 year
Malouf et al (2017)^79^	USA	Jail	49	31 (63%)	Males	37·2 years (range 18–81; SD 15·7)	Re-entry values and mindfulness programme plus treatment as usual; other; group	Treatment as usual	4 weeks total; 90 min sessions, twice per week	Rearrest	3 years
Gold et al (2020)^80^	Norway	Prison	66	64 (96%)	Males	Median 26 years (range 18–53; SD not reported)	Music therapy; other; usually group but in some cases individual	Standard care	Mean 4·4 (range 0–12; SD 3·9); median 3·0), typically two to three times per week	Serious events, excluding writs	5 years
Hein et al (2020)^49^	USA	Juvenile justice setting	289	289 (100%)	Males	14·9 years (SD 1·0)	Training on solving social problems; CBT-based; group	Treatment as usual	10 sessions each lasting 1 h	At least one offence during follow-up	2 years

Data are n (%) or mean (SD), unless otherwise specified. CBT=cognitive behavioural therapy.

In terms of risk of bias, most RCTs were rated as having concerns (n=18, 60%) or being at high risk (n=10, 33%), and only two studies^46^, ^54^ were rated as having a low risk of bias (appendix pp 10–12). There was a low risk of bias in outcome measurement for all studies, because recidivism was ascertained from official criminal records.

**Figure 2:**
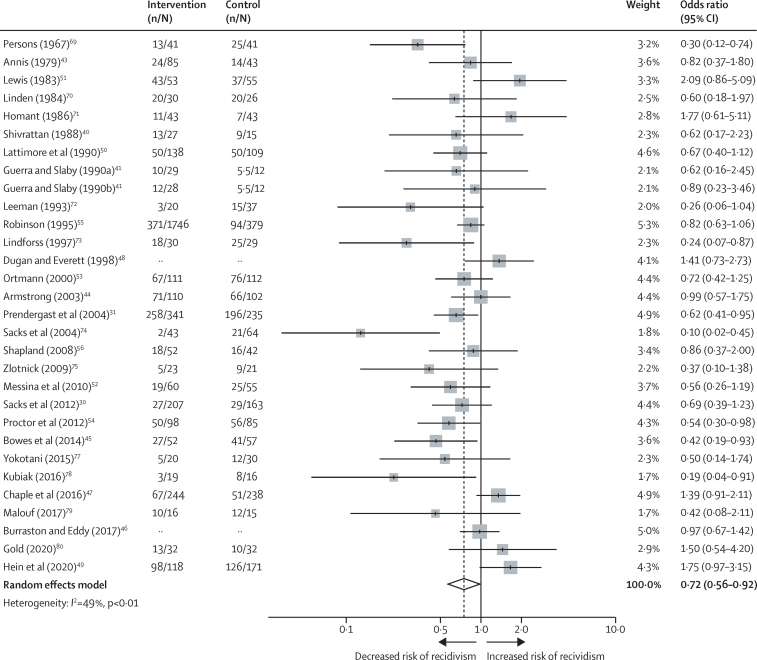
Effectiveness of psychological interventions in prison in reducing recidivism Data are for all 29 included randomised controlled trials. Error bars show 95% CI. The number of participants in the intervention and control groups were not available for Dugan and Everett^48^ or Burraston and Eddy^46^ because these studies presented outcomes as continuous rather than dichotomous data.

**Figure 3:**
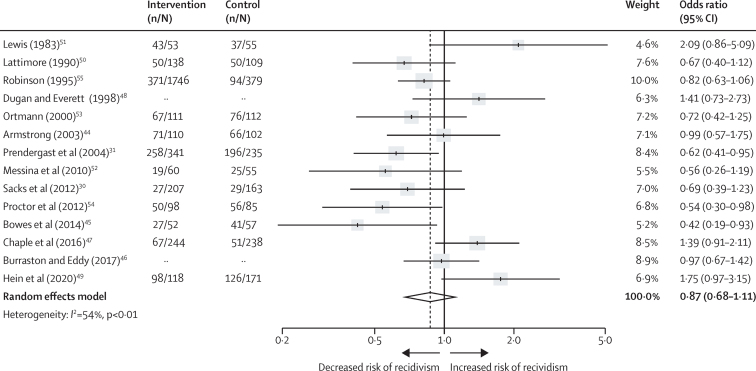
Effectiveness of psychological interventions in prison in reducing recidivism Data are for the 14 randomised controlled trials with an intervention group of at least 50 participants, excluding two outlier studies.^43^, ^56^ Error bars show 95% CI. The number of participants in the intervention and control groups were not available for Dugan and Everett^48^ or Burraston and Eddy^46^ because these studies presented outcomes as continuous rather than dichotomous data.

Overall in the meta-analysis, psychological interventions were associated with reduced reoffending, with a pooled OR of 0·72 (95% CI 0·56–0·92) and moderate levels of heterogeneity (*I*^2^=49%; *Q*=57·3; p<0·01; figure 2). To prevent overestimation caused by small-study effect, as suggested by the literature^65^, ^66^ and confirmed by our influence analysis, we pooled results excluding studies with fewer than 50 participants in the experimental group, as a planned sensitivity analysis. The reduction in recidivism was attenuated in the 14 trials (6446 followed-up participants) with an intervention group of at least 50 participants (OR 0·87, 95% CI 0·68–1·11; *I*^2^=54%; figure 3).

**Figure 4:**
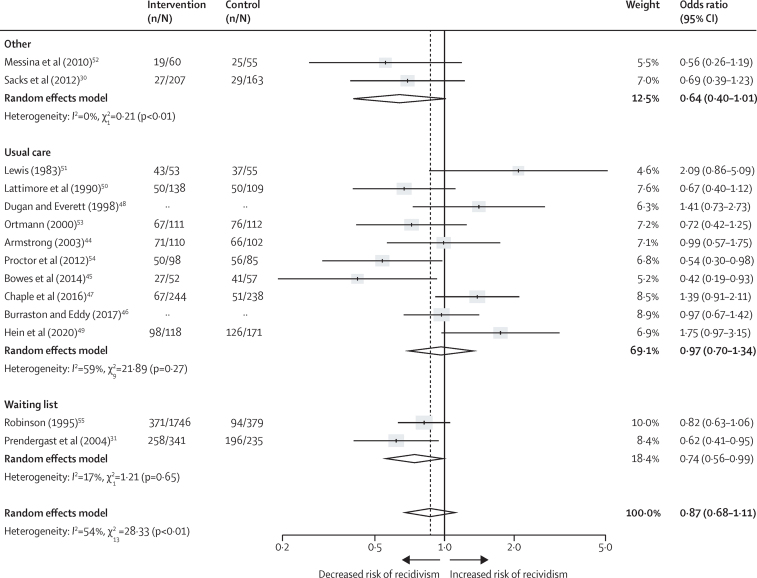
Effectiveness of psychological interventions in prison for reducing recidivism, by comparator type Data are for randomised controlled trials with an intervention group of at least 50 participants, excluding two outlier studies.^43^, ^56^ Error bars show 95% CI. The number of participants in the intervention and control groups were not available for Dugan and Everett^48^ or Burraston and Eddy^46^ because these studies presented outcomes as continuous rather than dichotomous data.

**Figure 5:**
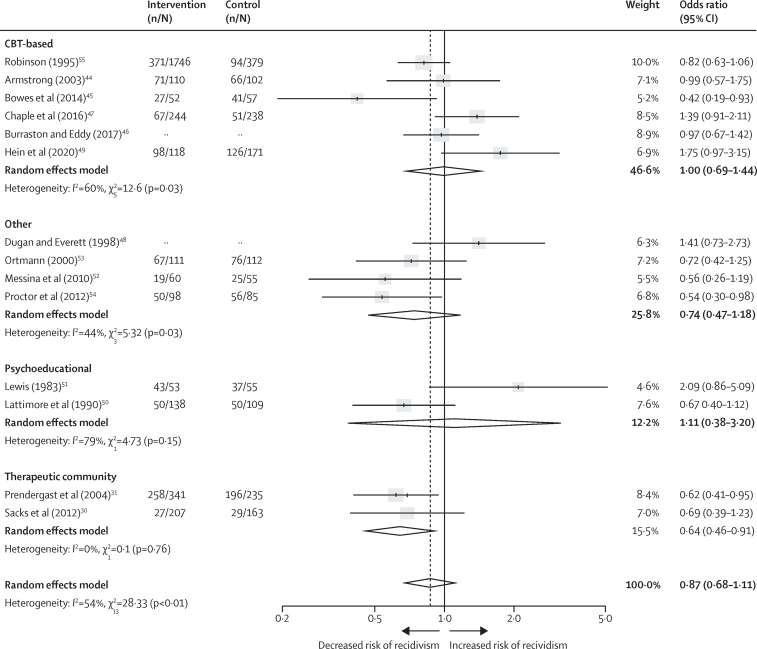
Effectiveness of psychological interventions in prison for reducing recidivism, by intervention type Data are for randomised controlled trials with an intervention group of at least 50 participants, excluding two outlier studies.^43^, ^56^ Error bars show 95% CI. CBT=cognitive behavioural therapy.

Subgroup analyses are shown by comparator type in figure 4, and by intervention type in figure 5. RCTs with a control group of usual care were associated with recidivism but not significantly so (OR 0·97, 95% CI 0·70–1·34; *I*^2^=59%). If using waiting list (0·74, 0·56–0·99; 17%) or other interventions (0·64, 0·40–1·01; 0%), the reduction in recidivism was larger although CIs were overlapping. By treatment modality, CBT-based interventions were not associated with recidivism (1·00, 0·69–1·44; 60%) neither were psychoeducational interventions (1·11, 0·38–3·20; 79%). Other types of interventions were associated with non-significant reductions in recidivism (0·74, 0·47–1·18; 44%). However, there were reductions in reoffending risk for therapeutic community programmes (0·64, 0·46–0·91; 0%).

On univariate analyses, there was a statistically significant difference between the pooled effects of trials which included sex-specific samples compared with trails that included both males and females (*Q* 4·30; p=0·04). Sex-specific interventions were significantly associated with reduced recidivism (OR 0·67, 95% CI 0·50–0·90), whereas those including both males and females were not (1·09, 0·77–1·55). No other significant associations were found between prespecified study characteristics and effect sizes in subgroup or meta-regression analyses (table 2).

**Table 2:** Meta-regression analyses assessing links between study characteristics and recidivism risk

	**β**	**SE**	**p value**
Year of publication: ≥1990 *vs* <1990	−0·195	0·335	0·560
Study location: USA *vs* elsewhere	0·097	0·274	0·722
Sample size (continuous)	0·000	0·000	0·671
Sex of participants: single sex *vs* both sexes	−0·404	0·371	0·276
Mean age (continuous)	−0·016	0·018	0·372
Age group: adolescents *vs* adults	−0·161	0·284	0·570
Intervention type: cognitive behavioural therapy-based *vs* all other types	−0·217	0·270	0·422
Comparator type: usual care *vs* waitlist or other	0·396	0·301	0·189
Follow-up time period (continuous)	0·074	0·063	0·239
Intervention format: individual *vs* group or combination	−0·055	0·348	0·875
Intervention aimed at people in prison with a substance use disorder (dichotomous)	−0·283	0·256	0·269
Risk of bias: high *vs* low or unclear	−0·146	0·266	0·583

Two studies^49^, ^74^ that contributed disproportionately to the pooled effect were identified using influence analyses in all RCTs. Removal of these outliers reduced the degree of heterogeneity between studies from moderate (*I*^2^=49%) to low (38%) but did not materially alter the pooled effect size (OR 0·73, 95% CI 0·58–0·91; appendix pp 13–15).

We found evidence of publication bias using Egger's test (t = –2·12; p=0·04) suggesting small-study effects. This finding was supported by visual inspection of the related funnel plot, which showed asymmetry (appendix pp 16–17). Seven smaller studies were identified and trimmed using the trim and fill method,^40^, ^69^, ^72^, ^73^, ^74^, ^75^, ^78^ and the OR after adjusting for publication bias was 0·86 (95% CI 0·65–1·15).

The fixed-effect estimate (OR 0·81, 95% CI 0·72–0·91; *I*^2^=49%; appendix p 18) did not materially differ from the random-effects model. Repeating the meta-analysis and only including larger studies (ie, ≥100 participants in the psychological intervention group) resulted in a decrease of the strength of the association to OR 0·90 (0·71–1·14; appendix p 19).^60^

## Discussion

In this meta-analysis of psychological interventions for recidivism, we identified 29 jail-based or prison-based RCTs of 9443 individuals from seven countries. Overall, there was evidence of reduced odds of reoffending. To account for small-study effects, in a planned sensitivity analysis, we excluded studies with fewer than 50 people in each experimental arm, resulting in 14 trials with 6446 followed-up participants, and the overall pooled OR 0·87 (95% CI 0·68–1·11) indicated, at most, modest effects.

We report two other main findings. First, in a sensitivity analysis, we found no strong evidence of reduced reoffending after participation in CBT-based programmes in prison (OR 1·00, 95% CI 0·69–1·44; *I*^2^=60%). This is by contrast with a 2007 systematic review combining both prison-based and community-based interventions that reported reduced risks of 20–30%.^13^ One potential explanation for no clear effectiveness of such CBT interventions found in the current systematic review is that these interventions are not linked with psychosocial support upon release. It might also be that these psychological therapies, which were developed for mental health problems, do not address the accommodation, employment, and financial difficulties after release that contribute to recidivism risk.^81^

A second finding, from a subgroup analysis, was that participation in a therapeutic community was associated with reduced reoffending risk. However, this finding was limited to only two studies,^30^, ^31^ both of which linked people released from prison to voluntary post-prison services. In support of this finding, in one of the two trials, links to community services were associated with a lower return to custody rate (33 [42%] of 79) than for participants without such links (137 [86%] of 159).^31^ Findings from a systematic review^82^ of psychoeducational programmes for reducing prison violence are consistent with the potential role of therapeutic communities, as programmes tailored to specific needs (eg, substance use disorder) were associated with reduced institutional violence. Similar results were reported in a Cochrane review^83^ of any people who offended and had co-occurring drug and mental health problems, as three^35^, ^74^, ^76^ of the four included studies^35^, ^56^, ^74^, ^76^ found therapeutic communities were associated with reductions in recidivism.

There are several implications for treatments offered in prison. First, in-prison interventions might not be effective unless they are linked with interventions that target the psychosocial needs of released individuals. For example, two therapeutic community trials^30^, ^31^ highlighted the potential importance of community aftercare to maintain the therapeutic gains delivered in prison. Hence, psychological interventions that combine prison-based and community-based services should be prioritised for future research. It should be noted that UK efforts to implement the Through the Gate service for resettling people released from prison have been widely criticised for inadequate communication between prisons and community services, and for poor assessment of resettlement needs, which should occur early in the sentence of a person in prison.^84^

Second, most of the tested interventions were developed in the community or in clinical populations for other outcomes, and hence might not address risk factors specific to reoffending. Such risk factors need to be identified by high quality assessment, and then linked to interventions for reducing recidivism. Risk assessments should be informed by scalable and transparent clinical prediction tools, such as the Oxford Risk of Recidivism tool (also known as OxRec),^85^ which includes assessment of modifiable risk factors for recidivism (eg, substance misuse and mental health status), supplemented by detailed assessments that consider additional dynamic factors. Considering that the resources allocated for interventions in prison populations are limited,^86^ stratification of risk is necessary to guide risk management and the treatment of people on release from prison.

A third implication regards CBT. The absence of effect that we reported is different to evidence from some reviews (including one published by the Campbell Collaboration^13^), which have suggested that CBT is one of the most effective forms of treatment for people in prison.^7^, ^8^, ^9^, ^10^, ^11^, ^12^ However, these previous reviews combined RCTs with less than rigorous study designs and the current new findings question the widespread roll-out of these treatment approaches in prisons. Only one^45^ of the six CBT studies^44–47,49,55^ in our systematic review reported significant reductions in reoffending. Other research, in selected populations of all people who have offended and also use drugs, also found little support for CBT.^83^, ^87^

Another implication of our review is that the effects of in-prison psychological interventions on recidivism appear to be smaller than those reported in previous meta-analyses, which have been estimated to be around 0·65 (95% CI 0·57–0·75).^24^ This difference is probably because the previous reviews included studies using weak research designs, such as quasi-experimental studies.^88^ A similar difference has been noted for psychotherapy effectiveness in depression, whereby overall effectiveness was overestimated in earlier meta-analyses because of inclusion of non-experimental designs.^57^

Our review highlights several evidence gaps. Further research is needed to determine whether generic psychological interventions are effective in specific groups of incarcerated populations, such as people living with mental disorders other than substance misuse. Research suggests that tailored individualised interventions are associated with better treatment outcomes.^89^ Furthermore, to improve transition to the community, future research should develop and evaluate the effects of follow-up treatments in the community after release. Greater consideration should be given to understanding the influence of environmental factors within prisons on treatment effects. Potential effects could be limited by the setting, because prisons are not primarily therapeutic environments and they prioritise security over health and rehabilitation needs.^90^ To better understand this possibility, research comparing the effectiveness of the same treatment modality in prison versus in a community setting could provide information on whether the prison environment sustains behavioural change and what adaptations could improve treatment effectiveness in prisons.

To our best knowledge, we report the first meta-analysis of RCTs on the effectiveness of psychological interventions delivered in prisons for recidivism outcomes. Some limitations should be noted. The study selection process leading up to the full-text screening stage was done by a single reviewer. The included trials were delivered in high-income countries. In addition, the number of included studies was not large (n=29), which underlines the legal, practical, and ethical challenges of doing high-quality research in prisons.^58^, ^90^, ^91^ One specific problem encountered in doing clinical research in these settings is high dropout rates, which often result in small and selective samples. Prisons have high turnover rates and participants are likely to be released or transferred unexpectedly.^92^ Furthermore, despite limiting inclusion to the most robust study design of RCT, only two (7%) of 29 of the included studies had low risk of bias. The most affected domains were randomisation and deviations from the intended interventions. Difficulties associated with masking staff and participants to the assigned intervention are likely to have contributed to an increased risk of bias in these two domains. There was also evidence of selective publication of small studies on the basis of their effect size (ie, some studies with small effect sizes were missing), which indicated that our initial pooled estimate of all studies (OR 0·72) was overestimated because of publication bias.^93^ Sex-specific analyses comparing estimates in females and males could not be done, because of insufficient numbers of female-only samples.

In conclusion, we have provided a synthesis of current research on the effectiveness of psychological interventions delivered in prisons aimed at reducing post-release recidivism. We report modest effects, at best, for psychological interventions delivered in prison. Trials of therapeutic community interventions and related approaches that facilitate continuity of treatment after prison release should be prioritised. Considering high rates of recidivism^3^, ^4^ and the consequences for public health and safety,^5^, ^6^ simple, large RCTs on the effectiveness of psychological interventions in prison are necessary.

## Data sharing

Data are based on the results of published studies listed in the appendix and available online. The study protocol and statistical analysis plan are available online.

For the **study protocol and statistical analysis plan** see https://www.crd.york.ac.uk/prospero/display_record.php?RecordID=167228

For more on the **Cochrane Collaboration's risk-of-bias tool for randomised trials (RoB 2)** see https://methods.cochrane.org/bias/resources/rob-2-revised-cochrane-risk-bias-tool-randomized-trials

## Declaration of interests

We declare no competing interests.

## References

[1] UN Office on Drugs and Crime, International Labour Organization, UNDP, WHO, UNAIDS (June, 2013). HIV prevention, treatment and care in prisons and other closed settings: a comprehensive package of interventions. https://www.who.int/hiv/pub/prisons/prisons_package.pdf?ua=1.

[2] Walmsley R (September, 2018). World prison population list. https://www.prisonstudies.org/sites/default/files/resources/downloads/wppl_12.pdf.

[3] Petersilia J (2011). Beyond the prison bubble. Wilson Q.

[4] Yukhnenko D, Sridhar S, Fazel S (2020). A systematic review of criminal recidivism rates worldwide: 3-year update. Wellcome Open Res.

[5] Newton A, May X, Eames S, Ahmad M (2019). Economic and social costs of reoffending. Ministry of Justice Analytical Series. https://assets.publishing.service.gov.uk/government/uploads/system/uploads/attachment_data/file/814650/economic-social-costs-reoffending.pdf.

[6] Sentencing Policy Advisory Council (2018). Illinois results first: the High Cost of Recidivism 2018 report. https://spac.icjia-api.cloud/uploads/Illinois_Result_First-The_High_Cost_of_Recidivism_2018-20191106T18123262.pdf.

[7] Henwood KS, Chou S, Browne KD (2015). A systematic review and meta-analysis on the effectiveness of CBT informed anger management. Aggress Violent Behav.

[8] Landenberger NA, Lipsey MW (2005). The positive effects of cognitive–behavioral programs for offenders: a meta-analysis of factors associated with effective treatment. J Exp Criminol.

[9] Wilson DB, Bouffard LA, MacKenzie DL (2005). A quantitative review of structured, group-oriented, cognitive-behavioral programs for offenders. Crim Justice Behav.

[10] Usher AM, Stewart LA (2014). Effectiveness of correctional programs with ethnically diverse offenders: a meta-analytic study. Int J Offender Ther Comp Criminol.

[11] Lösel F, Schmucker M (2005). The effectiveness of treatment for sexual offenders: a comprehensive meta-analysis. J Exp Criminol.

[12] Pearson FS, Lipton DS, Cleland CM, Yee DS (2002). The effects of behavioral/cognitive-behavioral programs on recidivism. Crime Delinq.

[13] Lipsey MW, Landenberger NA, Wilson SJ (2007). Effects of cognitive-behavioral programs for criminal offenders. Campbell Syst Rev.

[14] Andrews DA, Bonta J, Hoge RD (1990). Classification for effective rehabilitation: rediscovering psychology. Crim Justice Behav.

[15] Dowden C, Andrews DA (2000). Effective correctional treatment and violent reoffending: a meta-analysis. Can J Criminol.

[16] Andrews DA, Bonta J (2010). Rehabilitating criminal justice policy and practice. Psychol Public Policy Law.

[17] Hanson RK, Bourgon G, Helmus L, Hodgson S (2009). The principles of effective correctional treatment also apply to sexual offenders: a meta-analysis. Crim Justice Behav.

[18] Hopkin G, Evans-Lacko S, Forrester A, Shaw J, Thornicroft G (2018). Interventions at the transition from prison to the community for prisoners with mental illness: a systematic review. Adm Policy Ment Health.

[19] Jolliffe D, Farrington DP (2007). A systematic review of the national and international evidence on the effectiveness of interventions with violent offenders.

[20] Koehler JA, Lösel F, Akoensi TD, Humphreys DK (2013). A systematic review and meta-analysis on the effects of young offender treatment programs in Europe. J Exp Criminol.

[21] Långström N, Enebrink P, Laurén E-M, Lindblom J, Werkö S, Hanson RK (2013). Preventing sexual abusers of children from reoffending: systematic review of medical and psychological interventions. BMJ.

[22] Lipsey MW, Cullen FT (2007). The effectiveness of correctional rehabilitation: a review of systematic reviews. Annu Rev Law Soc Sci.

[23] Lipsey MW, Wilson D, Loeber RM, Farrington DP (1998). Effective intervention for serious juvenile offenders: a synthesis of research. Serious and violent juvenile offenders: risk factors and successful intervention.

[24] Papalia N, Spivak B, Daffern M, Ogloff JR (2019). A meta-analytic review of the efficacy of psychological treatments for violent offenders in correctional and forensic mental health settings. Clin Psychol Sci Pract.

[25] Perry AE, Woodhouse R, Neilson M (2016). Are non-pharmacological interventions effective in reducing drug use and criminality? A systematic and meta-analytical review with an economic appraisal of these interventions. Int J Environ Res Public Health.

[26] Tripodi SJ, Bledsoe SE, Kim JS, Bender K (2011). Effects of correctional-based programs for female inmates: a systematic review. Res Soc Work Pract.

[27] Papalia N, Spivak B, Daffern M, Ogloff JR (2020). Are psychological treatments for adults with histories of violent offending associated with change in dynamic risk factors? A meta-analysis of intermediate treatment outcomes. Crim Justice Behav.

[28] Schmucker M, Lösel F (2015). The effects of sexual offender treatment on recidivism: an international meta-analysis of sound quality evaluations. J Exp Criminol.

[29] Dowden C, Andrews DA (1999). What works for female offenders: a meta-analytic review. Crime Delinq.

[30] Sacks JY, McKendrick K, Hamilton Z (2012). A randomized clinical trial of a therapeutic community treatment for female inmates: outcomes at 6 and 12 months after prison release. J Addict Dis.

[31] Prendergast ML, Hall EA, Wexler HK, Melnick G, Cao Y (2004). Amity prison-based therapeutic community: 5-year outcomes. Prison J.

[32] Nielsen AL, Scarpitti FR, Inciardi JA (1996). Integrating the therapeutic community and work release for drug-involved offenders. The CREST Program. J Subst Abuse Treat.

[33] Farrell A (2000). Women, crime and drugs: testing the effect of therapeutic communities. Women Crim Justice.

[34] Moher D, Liberati A, Tetzlaff J, Altman DG (2009). Preferred reporting items for systematic reviews and meta-analyses: the PRISMA statement. Ann Intern Med.

[35] Wexler HK, De Leon G, Thomas G, Kressel D, Peters J (1999). The Amity prison TC evaluation: reincarceration outcomes. Crim Justice Behav.

[36] Wexler HK, Melnick G, Lowe L, Peters J (1999). Three-year reincarceration outcomes for Amity in-prison therapeutic community and aftercare in California. Prison J.

[37] Sterne JAC, Savović J, Page MJ (2019). RoB 2: a revised tool for assessing risk of bias in randomised trials. BMJ.

[38] Higgins J, Altman D, Sterne J (2011). Cochrane handbook for systematic reviews of interventions. Version 5.0.

[39] Kingston DA, Olver ME, McDonald J, Cameron C (2018). A randomised controlled trial of a cognitive skills programme for offenders with mental illness. Crim Behav Ment Health.

[40] Shivrattan JL (1988). Social interactional training and incarcerated juvenile delinquents. Can J Criminol.

[41] Guerra NG, Slaby RG (1990). Cognitive mediators of aggression in adolescent offenders: II. Intervention. Dev Psychol.

[42] von Hippel PT (2015). The heterogeneity statistic *I*^2^ can be biased in small meta-analyses. BMC Med Res Methodol.

[43] Annis HM (1979). Group treatment of incarcerated offenders with alcohol and drug problems: a controlled evaluation. Can J Criminol.

[44] Armstrong TA (2003). The effect of moral reconation therapy on the recidivism of youthful offenders: a randomized experiment. Crim Justice Behav.

[45] Bowes N, McMurran M, Evans C, Oatley G, Williams B, David S (2014). Treating alcohol-related violence: a feasibility study of a randomized controlled trial in prisons. J Forensic Psychiatry Psychol.

[46] Burraston BO, Eddy JM (2017). The moderating effect of living with a child before incarceration on postrelease outcomes related to a prison-based parent management training program. Smith Coll Stud Soc Work.

[47] Chaple M, Sacks S, McKendrick K (2016). A comparative study of the therapeutic education system for incarcerated substance-abusing offenders. Prison J.

[48] Dugan JR, Everett RS (1998). An experimental test of chemical dependency therapy for jail inmates. Int J Offender Ther Comp Criminol.

[49] Hein S, Weeland J, Square A (2020). Effectiveness of a social problem solving training in youth in detention or on probation: an RCT and pre-post community implementation. Int J Law Psychiatry.

[50] Lattimore PK, Witte AD, Baker JR (1990). Experimental assessment of the effect of vocational training on youthful property offenders. Eval Rev.

[51] Lewis RV (1983). Scared straight—California style: evaluation of the San Quentin squires program. Crim Justice Behav.

[52] Messina N, Grella CE, Cartier J, Torres S (2010). A randomized experimental study of gender-responsive substance abuse treatment for women in prison. J Subst Abuse Treat.

[53] Ortmann R (2000). The effectiveness of social therapy in prison— a randomized experiment. Crime Delinq.

[54] Proctor SL, Hoffmann NG, Allison S (2012). The effectiveness of interactive journaling in reducing recidivism among substance-dependent jail inmates. Int J Offender Ther Comp Criminol.

[55] Robinson D (1995). The impact of cognitive skills training on post-release recidivism among Canadian federal offenders.

[56] Shapland J, Atkinson A, Atkinson H (June, 2008). Does restorative justice affect reconviction. The fourth report from the evaluation of three schemes. https://restorativejustice.org.uk/sites/default/files/resources/files/Does%20restorative%20justice%20affect%20reconviction.pdf.

[57] Cuijpers P, van Straten A, Bohlmeijer E, Hollon SD, Andersson G (2010). The effects of psychotherapy for adult depression are overestimated: a meta-analysis of study quality and effect size. Psychol Med.

[58] Farrington DP, Welsh BC (2005). Randomized experiments in criminology: what have we learned in the last two decades?. J Exp Criminol.

[59] Welsh BC, Peel ME, Farrington DP, Elffers H, Braga AA (2011). Research design influence on study outcomes in crime and justice: a partial replication with public area surveillance. J Exp Criminol.

[60] Cuijpers P, Cristea IA (2016). How to prove that your therapy is effective, even when it is not: a guideline. Epidemiol Psychiatr Sci.

[61] Baujat B, Mahé C, Pignon JP, Hill C (2002). A graphical method for exploring heterogeneity in meta-analyses: application to a meta-analysis of 65 trials. Stat Med.

[62] Egger M, Davey Smith G, Schneider M, Minder C (1997). Bias in meta-analysis detected by a simple, graphical test. BMJ.

[63] Peters JL, Sutton AJ, Jones DR, Abrams KR, Rushton L (2007). Performance of the trim and fill method in the presence of publication bias and between-study heterogeneity. Stat Med.

[64] Duval S, Tweedie R (2000). Trim and fill: A simple funnel-plot-based method of testing and adjusting for publication bias in meta-analysis. Biometrics.

[65] Schwarzer G, Carpenter JR, Rücker G (2015). Meta-analysis with R.

[66] Turner RM, Bird SM, Higgins JPT (2013). The impact of study size on meta-analyses: examination of underpowered studies in Cochrane reviews. PLoS One.

[67] RStudio Team (2019). RStudio: integrated development for R.

[68] R Core Team (2020). R: a language and environment for statistical computing.

[69] Persons RW (1967). Relationship between psychotherapy with institutionalized boys and subsequent community adjustment. J Consult Psychol.

[70] Linden R, Perry L, Ayers D, Parlett TAA (1984). An evaluation of a prison education program. Can J Criminol.

[71] Homant RJ (1986). Ten years after: a follow-up of therapy effectiveness. J Offender Rehabil.

[72] Leeman LW, Gibbs JC, Fuller D (1993). Evaluation of a multi-component group treatment program for juvenile delinquents. Aggress Behav.

[73] Lindforss L, Magnusson D (1997). Solution-focused therapy in prison. Contemp Fam Ther.

[74] Sacks S, Sacks JY, McKendrick K, Banks S, Stommel J (2004). Modified TC for MICA offenders: crime outcomes. Behav Sci Law.

[75] Zlotnick C, Johnson J, Najavits LM (2009). Randomized controlled pilot study of cognitive-behavioral therapy in a sample of incarcerated women with substance use disorder and PTSD. Behav Ther.

[76] Sacks S, Chaple M, Sacks JY, McKendrick K, Cleland CM (2012). Randomized trial of a reentry modified therapeutic community for offenders with co-occurring disorders: crime outcomes. J Subst Abuse Treat.

[77] Yokotani K, Tamura K (2015). Effects of personalized feedback interventions on drug-related reoffending: a pilot study. Prev Sci.

[78] Kubiak S, Fedock G, Kim WJ, Bybee D (2016). Long-term outcomes of a RCT intervention study for women with violent crimes. J Soc Social Work Res.

[79] Malouf ET, Youman K, Stuewig J, Witt EA, Tangney JP (2017). A pilot RCT of a values-based mindfulness group intervention with jail inmates: evidence for reduction in post-release risk behavior. Mindfulness.

[80] Gold C, Due FB, Thieu EK, Hjørnevik K, Tuastad L, Assmus J (2020). Long-term effects of short-term music therapy for prison inmates: six-year follow-up of a randomized controlled trial. Int J Offender Ther Comp Criminol.

[81] Hirschtritt ME, Binder RL (2017). Interrupting the mental illness–incarceration–recidivism cycle. JAMA.

[82] Auty KM, Cope A, Liebling A (2017). Psychoeducational programs for reducing prison violence: a systematic review. Aggress Violent Behav.

[83] Perry AE, Martyn-St James M, Burns L (2019). Interventions for drug-using offenders with co-occurring mental health problems. Cochrane Database Syst Rev.

[84] HM Inspectorate of Probation and HM Inspectorate of Prisons (October, 2016). An inspection of Through the Gate resettlement services for short-term prisoners. https://www.justiceinspectorates.gov.uk/cjji/wp-content/uploads/sites/2/2016/09/Through-the-Gate.pdf.

[85] Fazel S, Chang Z, Fanshawe T (2016). Prediction of violent reoffending on release from prison: derivation and external validation of a scalable tool. Lancet Psychiatry.

[86] Petersilia J, Rosenfeld R, Bonnie RJ Parole, desistance from crime, and community integration. https://cdpsdocs.state.co.us/ccjj/Resources/Ref/NCR2007.pdf.

[87] Perry AE, Martyn-St James M, Burns L (2019). Interventions for female drug-using offenders. Cochrane Database Syst Rev.

[88] Weisburd D, Lum CM, Petrosino A (2001). Does research design affect study outcomes in criminal justice?. Ann Am Acad Pol Soc Sci.

[89] Fontanarosa J, Uhl S, Oyesanmi O, Schoelles KM (2013). Interventions for adult offenders with serious mental illness.

[90] Yoon IA, Slade K, Fazel S (2017). Outcomes of psychological therapies for prisoners with mental health problems: a systematic review and meta-analysis. J Consult Clin Psychol.

[91] Quina K, Garis AV, Stevenson J (2007). Through the bullet-proof glass: conducting research in prison settings. J Trauma Dissociation.

[92] Lobmaier PP, Kunøe N, Waal H (2010). Treatment research in prison: problems and solutions in a randomized trial. Addict Res Theory.

[93] Borenstein M, Hedges LV, Higgins JP, Rothstein HR (2011). Introduction to meta-analysis.

