# Supplementary material for: Effectiveness of psychological interventions in prison to reduce recidivism: a systematic review and meta-analysis of randomised controlled trials
Source: Lancet Psychiatry. 2021 Sep;8(9):759–73. doi: 10.1016/S2215-0366(21)00170-X (PMC8376657; doi:10.1016/S2215-0366(21)00170-X)
Supplement: Supplementary appendix [file mmc1.pdf]

THE LANCET
Psychiatry

Supplementary appendix

This appendix formed part of the original submission and has been peer reviewed. We post it as supplied by the authors.

Supplement to: Beaudry G, Yu R, Perry AE, Fazel S. Effectiveness of psychological interventions in prison to reduce recidivism: a systematic review and meta-analysis of randomised controlled trials. *Lancet Psychiatry* 2021; **8:** 759–73.

Table A1. PRISMA checklist

Section/topic	#	Checklist item	Reported on page #
**Title**			
Title	1	Identify the report as a systematic review, meta-analysis, or both.	1
**Abstract**			
Structured summary	2	Provide a structured summary including, as applicable: background; objectives; data sources; study eligibility criteria, participants, and interventions; study appraisal and synthesis methods; results; limitations; conclusions and implications of key findings; systematic review registration number.	1
**Introduction**			
Rationale	3	Describe the rationale for the review in the context of what is already known.	2
Objectives	4	Provide an explicit statement of questions being addressed with reference to participants, interventions, comparisons, outcomes, and study design (PICOS).	2
**Methods**			
Protocol and registration	5	Indicate if a review protocol exists, if and where it can be accessed (e.g., Web address), and, if available, provide registration information including registration number.	8
Eligibility criteria	6	Specify study characteristics (e.g., PICOS, length of follow-up) and report characteristics (e.g., years considered, language, publication status) used as criteria for eligibility, giving rationale.	2-3
Information sources	7	Describe all information sources (e.g., databases with dates of coverage, contact with study authors to identify additional studies) in the search and date last searched.	2
Search	8	Present full electronic search strategy for at least one database, including any limits used, such that it could be repeated.	3-7 (appendix)
Study selection	9	State the process for selecting studies (i.e., screening, eligibility, included in systematic review, and, if applicable, included in the meta-analysis).	3
Data collection process	10	Describe method of data extraction from reports (e.g., piloted forms, independently, in duplicate) and any processes for obtaining and confirming data from investigators.	3-4
Data items	11	List and define all variables for which data were sought (e.g., PICOS, funding sources) and any assumptions and simplifications made.	3-4
Risk of bias in individual studies	12	Describe methods used for assessing risk of bias of individual studies (including specification of whether this was done at the study or outcome level), and how this information is to be used in any data synthesis.	4 & 8
Summary measures	13	State the principal summary measures (e.g., risk ratio, difference in means).	3-4
Synthesis of results	14	Describe the methods of handling data and combining results of studies, if done, including measures of consistency (e.g., I^2) for each meta-analysis.	3-4 & 8
Risk of bias across studies	15	Specify any assessment of risk of bias that may affect the cumulative evidence (e.g., publication bias, selective reporting within studies).	8
Additional analyses	16	Describe methods of additional analyses (e.g., sensitivity or subgroup analyses, meta-regression), if done, indicating which were pre-specified.	3-4 & 8
**Results**			
Study selection	17	Give numbers of studies screened, assessed for eligibility, and included in the review, with reasons for exclusions at each stage, ideally with a flow diagram.	4
Study characteristics	18	For each study, present characteristics for which data were extracted (e.g., study size, PICOS, follow-up period) and provide the citations.	5-8
Risk of bias within studies	19	Present data on risk of bias of each study and, if available, any outcome level assessment (see item 12).	10-12 (appendix)
Results of individual studies	20	For all outcomes considered (benefits or harms), present, for each study: (a) simple summary data for each intervention group (b) effect estimates and confidence intervals, ideally with a forest plot.	9
Synthesis of results	21	Present results of each meta-analysis done, including confidence intervals and measures of consistency.	9-12

Risk of bias across studies	22	Present results of any assessment of risk of bias across studies (see Item 15).	9
Additional analysis	23	Give results of additional analyses, if done (e.g., sensitivity or subgroup analyses, meta-regression [see Item 16]).	9-12
**Discussion**			
Summary of evidence	24	Summarize the main findings including the strength of evidence for each main outcome; consider their relevance to key groups (e.g., healthcare providers, users, and policy makers).	12-13
Limitations	25	Discuss limitations at study and outcome level (e.g., risk of bias), and at review-level (e.g., incomplete retrieval of identified research, reporting bias).	13
Conclusions	26	Provide a general interpretation of the results in the context of other evidence, and implications for future research.	12-13
**Funding**			
Funding	27	Describe sources of funding for the systematic review and other support (e.g., supply of data); role of funders for the systematic review.	8 & 13

From: Moher D, Liberati A, Tetzlaff J, Altman DG, The PRISMA Group (2009). Preferred Reporting Items for Systematic Reviews and Meta-Analyses: The PRISMA Statement. PLoS Med 6(7): e1000097. doi:10.1371/journal.pmed1000097

Text A1. Search strategy.

The search strategies were initially run on June 24, 2019 by Elinor Harriss, librarian at the Bodleian Health Care Libraries, University of Oxford. They were subsequently updated on February 17, 2021 by Gabrielle Beaudry.

Medline (Ovid MEDLINE® Epub Ahead of Print, In-Process & Other Non-Indexed Citations, Ovid MEDLINE® Daily and Ovid MEDLINE®) 1946 to present

----

1 (Randomized Controlled Trial or Controlled Clinical Trial or Pragmatic Clinical Trial or Equivalence Trial or Clinical Trial, Phase III).pt. (615901)
2 Randomized Controlled Trial/ (522962)
3 exp Randomized Controlled Trials as Topic/ (143830)
4 "Randomized Controlled Trial (topic)"/ (0)
5 Controlled Clinical Trial/ (94068)
6 exp Controlled Clinical Trials as Topic/ (149298)
7 "Controlled Clinical Trial (topic)"/ (0)
8 Randomization/ (104661)
9 Random Allocation/ (104661)
10 Double-Blind Method/ (162383)
11 Double Blind Procedure/ (0)
12 Double-Blind Studies/ (162383)
13 Single-Blind Method/ (29726)
14 Single Blind Procedure/ (0)
15 Single-Blind Studies/ (29726)
16 Placebos/ (35331)
17 Placebo/ (0)
18 Control Groups/ (1718)
19 Control Group/ (1718)
20 (random* or sham or placebo*).ti,ab,hw,kw. (1570431)
21 ((singl* or doubl*) adj (blind* or dumm* or mask*)).ti,ab,hw,kw. (244156)
22 ((tripl* or trebl*) adj (blind* or dumm* or mask*)).ti,ab,hw,kw. (1137)
23 (control* adj3 (study or studies or trial* or group*)).ti,ab,kw. (1034249)
24 (Nonrandom* or non random* or non-random* or quasi-random* or quasirandom*).ti,ab,hw,kw. (46484)
25 allocated.ti,ab,hw. (69884)
26 ((open label or open-label) adj5 (study or studies or trial*)).ti,ab,hw,kw. (36928)
27 ((equivalence or superiority or non-inferiority or noninferiority) adj3 (study or studies or trial*)).ti,ab,hw,kw. (9184)
28 (pragmatic study or pragmatic studies).ti,ab,hw,kw. (441)
29 ((pragmatic or practical) adj3 trial*).ti,ab,hw,kw. (5689)
30 ((quasiexperimental or quasi-experimental) adj3 (study or studies or trial*)).ti,ab,hw,kw. (8685)
31 (phase adj3 (III or "3") adj3 (study or studies or trial*)).ti,hw,kw. (29714)
32 exp clinical study/ (982445)
33 (program* or intervention* or treatment* or therap* or trial*).ti,ab. (7868637)
34 1 or 2 or 3 or 4 or 5 or 6 or 7 or 8 or 9 or 10 or 11 or 12 or 13 or 14 or 15 or 16 or 17 or 18 or 19 or 20 or 21 or 22 or 23 or 24 or 25 or 26 or 27 or 28 or 29 or 30 or 31 or 32 or 33 (9014861)
35 prison/ (9972)
36 (prison* or incarcerat* or custod* or imprison* or detain* or inmate* or jail* or gaol* or penal* or penitentiary or "correctional facilit*" or probation*).ti,ab. (55114)
37 35 or 36 (58191)
38 recidivism/ (368)
39 (recommit* or re-commit* or reoffend* or re-offend* or recidiv* or "repeat offen*" or reimprison* or re-imprison* or reincarcerat* or re-incarcerat* or reconvict* or re-convict* or rearrest* or re-arrest*).ti,ab. (7036)
40 38 or 39 (7086)
41 34 and 37 and 40 (1167)
42 exp experimental design/ (454407)

43 exp prisons/ (10539)
44 36 or 43 (58640)
45 34 or 42 (9106981)
46 40 and 44 and 45 (1172)

Embase 1974 to present

----

1 (Randomized Controlled Trial or Controlled Clinical Trial or Pragmatic Clinical Trial or Equivalence Trial or Clinical Trial, Phase III).pt. (0)
2 Randomized Controlled Trial/ (649240)
3 exp Randomized Controlled Trials as Topic/ (197072)
4 "Randomized Controlled Trial (topic)"/ (197072)
5 Controlled Clinical Trial/ (469590)
6 exp Controlled Clinical Trials as Topic/ (204974)
7 "Controlled Clinical Trial (topic)"/ (11472)
8 Randomization/ (90411)
9 Random Allocation/ (86596)
10 Double-Blind Method/ (158182)
11 Double Blind Procedure/ (182720)
12 Double-Blind Studies/ (141482)
13 Single-Blind Method/ (40029)
14 Single Blind Procedure/ (42056)
15 Single-Blind Studies/ (42056)
16 Placebos/ (308772)
17 Placebo/ (364888)
18 Control Groups/ (110544)
19 Control Group/ (110544)
20 (random* or sham or placebo*).ti,ab,hw,kw. (2164252)
21 ((singl* or doubl*) adj (blind* or dumm* or mask*)).ti,ab,hw,kw. (319913)
22 ((tripl* or trebl*) adj (blind* or dumm* or mask*)).ti,ab,hw,kw. (1502)
23 (control* adj3 (study or studies or trial* or group*)).ti,ab,kw. (1450043)
24 (Nonrandom* or non random* or non-random* or quasi-random* or quasirandom*).ti,ab,hw,kw. (58495)
25 allocated.ti,ab,hw. (90765)
26 ((open label or open-label) adj5 (study or studies or trial*)).ti,ab,hw,kw. (68177)
27 ((equivalence or superiority or non-inferiority or noninferiority) adj3 (study or studies or trial*)).ti,ab,hw,kw. (13712)
28 (pragmatic study or pragmatic studies).ti,ab,hw,kw. (660)
29 ((pragmatic or practical) adj3 trial*).ti,ab,hw,kw. (6136)
30 ((quasiexperimental or quasi-experimental) adj3 (study or studies or trial*)).ti,ab,hw,kw. (13882)
31 (phase adj3 (III or "3") adj3 (study or studies or trial*)).ti,hw,kw. (98236)
32 exp clinical study/ (10346360)
33 (program* or intervention* or treatment* or therap* or trial*).ti,ab. (10542184)
34 1 or 2 or 3 or 4 or 5 or 6 or 7 or 8 or 9 or 10 or 11 or 12 or 13 or 14 or 15 or 16 or 17 or 18 or 19 or 20 or 21 or 22 or 23 or 24 or 25 or 26 or 27 or 28 or 29 or 30 or 31 or 32 or 33 (17192461)
35 prison/ (15593)
36 (prison* or incarcerat* or custod* or imprison* or detain* or inmate* or jail* or gaol* or penal* or penitentiary or "correctional facilit*" or probation*).ti,ab. (65520)
37 35 or 36 (69306)
38 recidivism/ (3773)
39 (recommit* or re-commit* or reoffend* or re-offend* or recidiv* or "repeat offen*" or reimprison* or re-imprison* or reincarcerat* or re-incarcerat* or reconvict* or re-convict* or rearrest* or re-arrest*).ti,ab. (8948)
40 38 or 39 (9746)
41 34 and 37 and 40 (1787)

PsycINFO 1806 to present

----

1 (Randomized Controlled Trial or Controlled Clinical Trial or Pragmatic Clinical Trial or Equivalence Trial or Clinical Trial, Phase III).pt. (0)
2 Randomized Controlled Trial/ (657)
3 exp Randomized Controlled Trials as Topic/ (0)
4 "Randomized Controlled Trial (topic)"/ (0)
5 Controlled Clinical Trial/ (0)
6 exp Controlled Clinical Trials as Topic/ (0)
7 "Controlled Clinical Trial (topic)"/ (0)
8 Randomization/ (0)
9 Random Allocation/ (0)
10 Double-Blind Method/ (0)
11 Double Blind Procedure/ (0)
12 Double-Blind Studies/ (0)
13 Single-Blind Method/ (0)
14 Single Blind Procedure/ (0)
15 Single-Blind Studies/ (0)
16 Placebos/ (0)
17 Placebo/ (5907)
18 Control Groups/ (925)
19 Control Group/ (925)
20 [(random* or sham or placebo*).ti,ab,hw,kw.] (0)
21 [((singl* or doubl*) adj (blind* or dumm* or mask*)).ti,ab,hw,kw.] (0)
22 [((tripl* or trebl*) adj (blind* or dumm* or mask*)).ti,ab,hw,kw.] (0)
23 [(control* adj3 (study or studies or trial* or group*)).ti,ab,kw.] (0)
24 [(Nonrandom* or non random* or non-random* or quasi-random* or quasirandom*).ti,ab,hw,kw.] (0)
25 allocated.ti,ab,hw. (11111)
26 [((open label or open-label) adj5 (study or studies or trial*)).ti,ab,hw,kw.] (0)
27 [((equivalence or superiority or non-inferiority or noninferiority) adj3 (study or studies or trial*)).ti,ab,hw,kw.] (0)
28 [(pragmatic study or pragmatic studies).ti,ab,hw,kw.] (0)
29 [((pragmatic or practical) adj3 trial*).ti,ab,hw,kw.] (0)
30 [((quasiexperimental or quasi-experimental) adj3 (study or studies or trial*)).ti,ab,hw,kw.] (0)
31 [(phase adj3 (III or "3") adj3 (study or studies or trial*)).ti,hw,kw.] (0)
32 exp clinical study/ (0)
33 (program* or intervention* or treatment* or therap* or trial*).ti,ab. (1438553)
34 1 or 2 or 3 or 4 or 5 or 6 or 7 or 8 or 9 or 10 or 11 or 12 or 13 or 14 or 15 or 16 or 17 or 18 or 19 or 20 or 21 or 22 or 23 or 24 or 25 or 26 or 27 or 28 or 29 or 30 or 31 or 32 or 33 (1444967)
35 prison/ (0)
36 (prison* or incarcerat* or custod* or imprison* or detain* or inmate* or jail* or gaol* or penal* or penitentiary or "correctional facilit*" or probation*).ti,ab. (57197)
37 35 or 36 (57197)
38 recidivism/ (6122)
39 (recommit* or re-commit* or reoffend* or re-offend* or recidiv* or "repeat offen*" or reimprison* or re-imprison* or reincarcerat* or re-incarcerat* or reconvict* or re-convict* or rearrest* or re-arrest*).ti,ab. (10723)
40 38 or 39 (11552)
41 34 and 37 and 40 (2918)
42 exp experimental design/ (58966)
43 exp prisons/ (7320)
44 36 or 43 (57440)
45 34 or 42 (1477872)
46 40 and 44 and 45 (2976)

Global Health <1973 to 2021 Week 06>

----

1 (Randomized Controlled Trial or Controlled Clinical Trial or Pragmatic Clinical Trial or Equivalence Trial or Clinical Trial, Phase III).pt. (0)
2 Randomized Controlled Trial/ (43800)
3 exp Randomized Controlled Trials as Topic/ (0)
4 "Randomized Controlled Trial (topic)"/ (0)
5 Controlled Clinical Trial/ (0)
6 exp Controlled Clinical Trials as Topic/ (0)
7 "Controlled Clinical Trial (topic)"/ (0)
8 Randomization/ (0)
9 Random Allocation/ (0)
10 Double-Blind Method/ (0)
11 Double Blind Procedure/ (0)
12 Double-Blind Studies/ (0)
13 Single-Blind Method/ (0)
14 Single Blind Procedure/ (0)
15 Single-Blind Studies/ (0)
16 Placebos/ (1927)
17 Placebo/ (1927)
18 Control Groups/ (0)
19 Control Group/ (0)
20 [(random* or sham or placebo*).ti,ab,hw,kw.] (0)
21 [((singl* or doubl*) adj (blind* or dumm* or mask*)).ti,ab,hw,kw.] (0)
22 [((tripl* or trebl*) adj (blind* or dumm* or mask*)).ti,ab,hw,kw.] (0)
23 [(control* adj3 (study or studies or trial* or group*)).ti,ab,kw.] (0)
24 [(Nonrandom* or non random* or non-random* or quasi-random* or quasirandom*).ti,ab,hw,kw.] (0)
25 allocated.ti,ab,hw. (12065)
26 [((open label or open-label) adj5 (study or studies or trial*)).ti,ab,hw,kw.] (0)
27 [((equivalence or superiority or non-inferiority or noninferiority) adj3 (study or studies or trial*)).ti,ab,hw,kw.] (0)
28 [(pragmatic study or pragmatic studies).ti,ab,hw,kw.] (0)
29 [((pragmatic or practical) adj3 trial*).ti,ab,hw,kw.] (0)
30 [((quasiexperimental or quasi-experimental) adj3 (study or studies or trial*)).ti,ab,hw,kw.] (0)
31 [(phase adj3 (III or "3") adj3 (study or studies or trial*)).ti,hw,kw.] (0)
32 exp clinical study/ (0)
33 (program* or intervention* or treatment* or therap* or trial*).ti,ab. (1138544)
34 1 or 2 or 3 or 4 or 5 or 6 or 7 or 8 or 9 or 10 or 11 or 12 or 13 or 14 or 15 or 16 or 17 or 18 or 19 or 20 or 21 or 22 or 23 or 24 or 25 or 26 or 27 or 28 or 29 or 30 or 31 or 32 or 33 (1144983)
35 prison/ (0)
36 (prison* or incarcerat* or custod* or imprison* or detain* or inmate* or jail* or gaol* or penal* or penitentiary or "correctional facilit*" or probation*).ti,ab. (8586)
37 35 or 36 (8586)
38 recidivism/ (0)
39 (recommit* or re-commit* or reoffend* or re-offend* or recidiv* or "repeat offen*" or reimprison* or re-imprison* or reincarcerat* or re-incarcerat* or reconvict* or re-convict* or rearrest* or re-arrest*).ti,ab. (474)
40 38 or 39 (474)
41 34 and 37 and 40 (114)
42 exp experimental design/ (1707)
43 exp prisons/ (2523)
44 36 or 43 (8690)
45 34 or 42 (1145956)
46 40 and 44 and 45 (115)

Cochrane Database of Systematic Reviews
Cochrane Central Register of Controlled Trials

----

1 MeSH descriptor: [Prisons] explode all trees (126)
2 (prison* or incarcerat* or custod* or imprison* or detain* or inmate* or jail* or gaol* or penal* or penitentiary or "correctional facilit*" or probation*) (3538)
3 #1 or #2 (3539)
4 MeSH descriptor: [Recidivism] explode all trees (20)
5 (recommit* or re-commit* or reoffend* or re-offend* or recidiv* or "repeat offen*" or reimprison* or re-imprison* or reincarcerat* or re-incarcerat* or reconvict* or re-convict* or rearrest* or re-arrest*) (1608)
6 #4 or #5 (1608)
7 #3 and #6 (295)

(41 Cochrane Reviews; 250 Trials)

Google Scholar

We searched Google Scholar for articles which had cited the included studies. However, no additional study was identified.

Search Results (February 17, 2021)

Ovid Medline	1172
Ovid Embase	1787
Ovid PsycINFO	2976
Ovid Global Health	115
Cochrane Database of Systematic Reviews and Cochrane CENTRAL	295
Google Scholar	0
Total	6345
Total (after deduplication)	4168

Table A2. Ethnicity data for each study

Study	Asian	Black/ African American	White	Hispanic/ Latinx	Indigenous	Other
**Person 1967**		40%	80%			
**Annis 1979**			89%			11%
**Lewis 1983**						
**Linden 1984**						
**Homant 1986**						
**Shivrattan 1988**						
**Lattimore 1990**			53%			47%
**Guerra 1990**			40%			60%
**Leeman 1993**		32%	67%	2%		
**Robinson 1995**					12%	
**Lindforss 1997**						
**Dugan 1998**		2%	72%	22%	4%	
**Ortmann 2000**						
**Armstrong 2003**	7%	55%	32%	6%		
**Prendergast 2004**		30%	38%	22%		10%
**Sacks 2004**		30%	49%	17%		4%
**Shapland 2008**						
**Zlotnick 2009**		33%	47%	14%		6%
**Messina 2010**		17%	48%	26%		9%
**Proctor 2012**		24%	73%			3%
**Sacks 2012**			47%	26%		
**Bowes 2014**	2%	2%	93%			3%

**Yokotani 2015**						
**Kubiak 2016**		54%	46%			
**Chaple 2016**		22%	49%	13%		17%
**Malouf 2017**		48%	27%	15%		10%
**Burraston 2017**		13%	59%	8%	8%	11%
**Gold 2020**						
**Hein 2020**		48%	17%	33%		2%

Note. This table is based on the race and ethnicity data presented in each individual study, and thus not all studies amount to 100%.

Table A3. Risk of bias assessment for each included study

Study	D1	D2	D3	D4	D5	Overall	Weight
**Person 1967**	Some concerns	Some concerns	Low	Low	Low	Some concerns	3.2
**Annis 1979**	Some concerns	Some concerns	Low	Low	Low	Some concerns	3.6
**Lewis 1983**	High	Some concerns	Low	Low	Low	High risk of bias	3.3
**Linden 1984**	High	Some concerns	Low	Low	High	High risk of bias	2.5
**Homant 1986**	Some concerns	Some concerns	Low	Low	High	High risk of bias	2.8
**Shivrattan 1988**	Some concerns	Some concerns	Low	Low	Low	Some concerns	2.3
**Lattimore 1990**	Some concerns	Some concerns	Low	Low	Low	Some concerns	4.6
**Guerra 1990a**	Some concerns	Some concerns	Low	Low	Low	Some concerns	2.1
**Guerra 1990b**	Some concerns	Some concerns	Low	Low	Low	Some concerns	2.1
**Leeman 1993**	Some concerns	Some concerns	Low	Low	Low	Some concerns	2.0
**Robinson 1995**	Some concerns	High	Low	Low	Low	High risk of bias	5.3
**Lindforss 1997**	Some concerns	Some concerns	Low	Low	Some concerns	High risk of bias	2.3
**Dugan 1998**	Some concerns	Some concerns	Low	Low	Low	Some concerns	4.1
**Ortmann 2000**	Some concerns	Some concerns	Low	Low	Some concerns	High risk of bias	4.4
**Armstrong 2003**	Some concerns	High	High	Low	Some concerns	High risk of bias	4.4
**Prendergast 2004**	High	Some concerns	Low	Low	Low	High risk of bias	4.9
**Sacks 2004**	Some concerns	Some concerns	Low	Low	Low	Some concerns	1.8
**Shapland 2008**	Some concerns	Some concerns	Low	Low	Low	Some concerns	3.4
**Zlotnick 2009**	Some concerns	Low	Low	Low	Low	Some concerns	2.2
**Messina 2010**	Some concerns	Low	Low	Low	Low	Some concerns	3.7
**Proctor 2012**	Low	Low	Low	Low	Low	Low risk of bias	4.3
**Sacks 2012**	Some concerns	Some concerns	Low	Low	Low	Some concerns	4.4
**Bowes 2014**	Some concerns	Low	Low	Low	Low	Some concerns	3.6
**Yokotani 2015**	Some concerns	Low	Low	Low	Low	Some concerns	2.3
**Kubiak 2016**	High	Some concerns	Low	Low	Low	High risk of bias	1.7
**Chaple 2016**	Some concerns	Low	Low	Low	Low	Some concerns	4.9

**Malouf 2017**	Low	High	Low	Low	Low	High risk of bias	1.7
**Burraston 2017**	Low	Low	Low	Low	Low	Low risk of bias	5.0
**Gold 2020**	Some concerns	Some concerns	Low	Low	Low	Some concerns	2.9
**Hein 2020**	Low	Some concerns	Low	Low	Low	Some concerns	4.3

Note. D1 = Bias arising from the randomization process; D2 = Bias due to deviations from intended interventions; D3 = Bias due to missing outcome data; D4 = Bias in measurement of the outcome; D5 = Bias in selection of the reported result.

Figure A1. Risk of bias summary bar plot

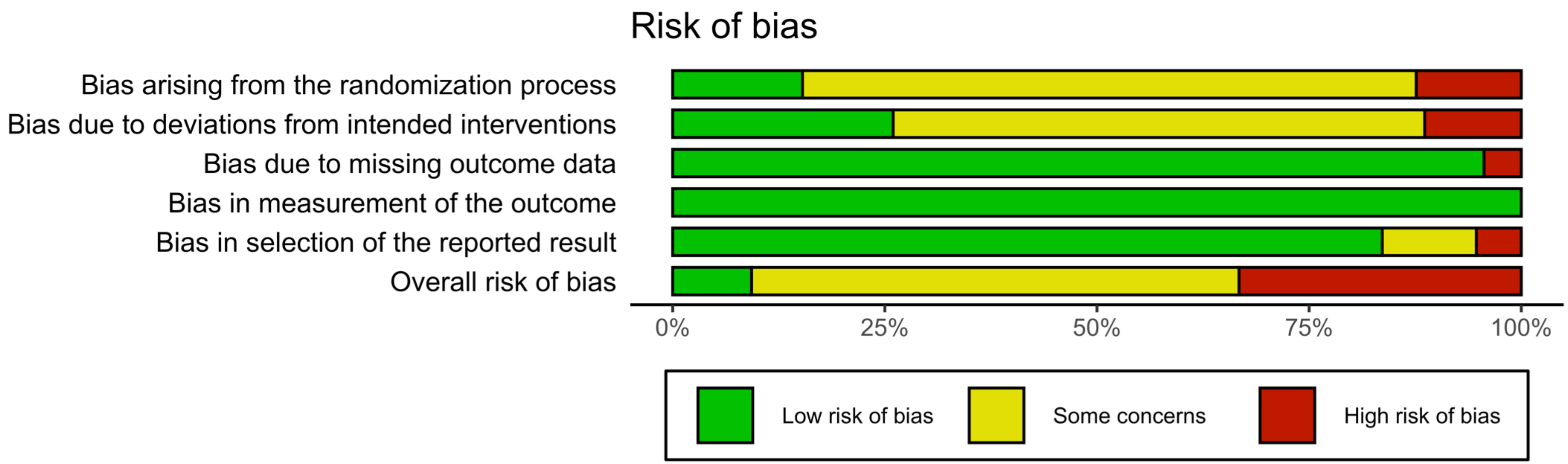

Figure A2. Forest plot of all studies with two outliers removed (random-effects model).

Study	TE	seTE	OR	95% CI	Weight
Persons 1967	−1.21	0.4634	0.30	[0.12; 0.74]	3.3%
Annis 1979	−0.20	0.4046	0.82	[0.37; 1.80]	3.8%
Lewis 1983	0.74	0.4536	2.09	[0.86; 5.09]	3.4%
Linden 1984	−0.51	0.6057	0.60	[0.18; 1.97]	2.4%
Homant 1986	0.57	0.5412	1.77	[0.61; 5.11]	2.8%
Shivrattan 1988	−0.48	0.6531	0.62	[0.17; 2.23]	2.2%
Lattimore 1990	−0.40	0.2612	0.67	[0.40; 1.12]	5.2%
Guerra 1990a	−0.47	0.6990	0.62	[0.16; 2.45]	2.0%
Guerra 1990b	−0.12	0.6945	0.89	[0.23; 3.46]	2.0%
Leeman 1993	−1.35	0.7115	0.26	[0.06; 1.04]	1.9%
Robinson 1995	−0.20	0.1326	0.82	[0.63; 1.06]	6.4%
Lindforss 1997	−1.43	0.6567	0.24	[0.07; 0.87]	2.2%
Dugan 1998	0.34	0.3363	1.41	[0.73; 2.73]	4.4%
Ortmann 2000	−0.33	0.2807	0.72	[0.42; 1.25]	5.0%
Armstrong 2003	−0.01	0.2877	0.99	[0.57; 1.75]	4.9%
Prendergast 2004	−0.48	0.2161	0.62	[0.41; 0.95]	5.6%
Sacks 2004	−2.30	0.7716	0.10	[0.02; 0.45]	0.0%
Shapland 2008	−0.15	0.4315	0.86	[0.37; 2.00]	3.6%
Zlotnick 2009	−0.99	0.6719	0.37	[0.10; 1.38]	2.1%
Messina 2010	−0.59	0.3878	0.56	[0.26; 1.19]	3.9%
Sacks 2012	−0.37	0.2907	0.69	[0.39; 1.23]	4.9%
Proctor 2012	−0.62	0.3048	0.54	[0.30; 0.98]	4.7%
Bowes 2014	−0.87	0.4044	0.42	[0.19; 0.93]	3.8%
Yokotani 2015	−0.69	0.6360	0.50	[0.14; 1.74]	2.3%
Kubiak 2016	−1.67	0.8024	0.19	[0.04; 0.91]	1.6%
Chaple 2016	0.33	0.2133	1.39	[0.91; 2.11]	5.7%
Malouf 2017	−0.87	0.8280	0.42	[0.08; 2.11]	1.5%
Burraston 2017	−0.03	0.1920	0.97	[0.67; 1.42]	5.9%
Gold 2020	0.41	0.5242	1.50	[0.54; 4.20]	2.9%
Hein 2020	0.56	0.3005	1.75	[0.97; 3.15]	0.0%
**Random effects model**			**0.73**	**[0.58; 0.91]**	**100.0%**

Heterogeneity: $I^2 = 38\%$, $p = 0.02$

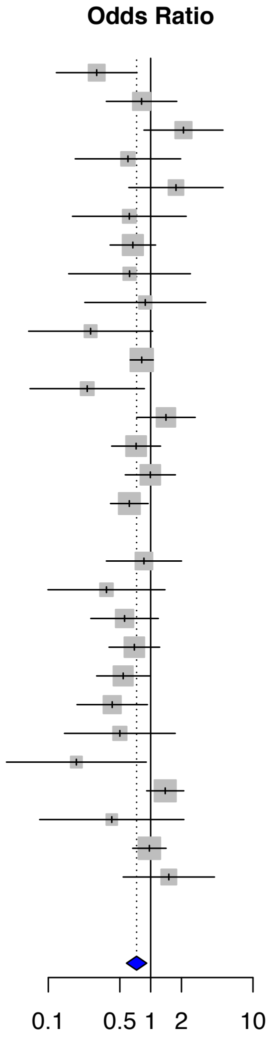

Note. The two removed outliers are Sacks 2004 and Hein 2020.

Figure A3. Leave-One-Out analyses (sorted by effect size).

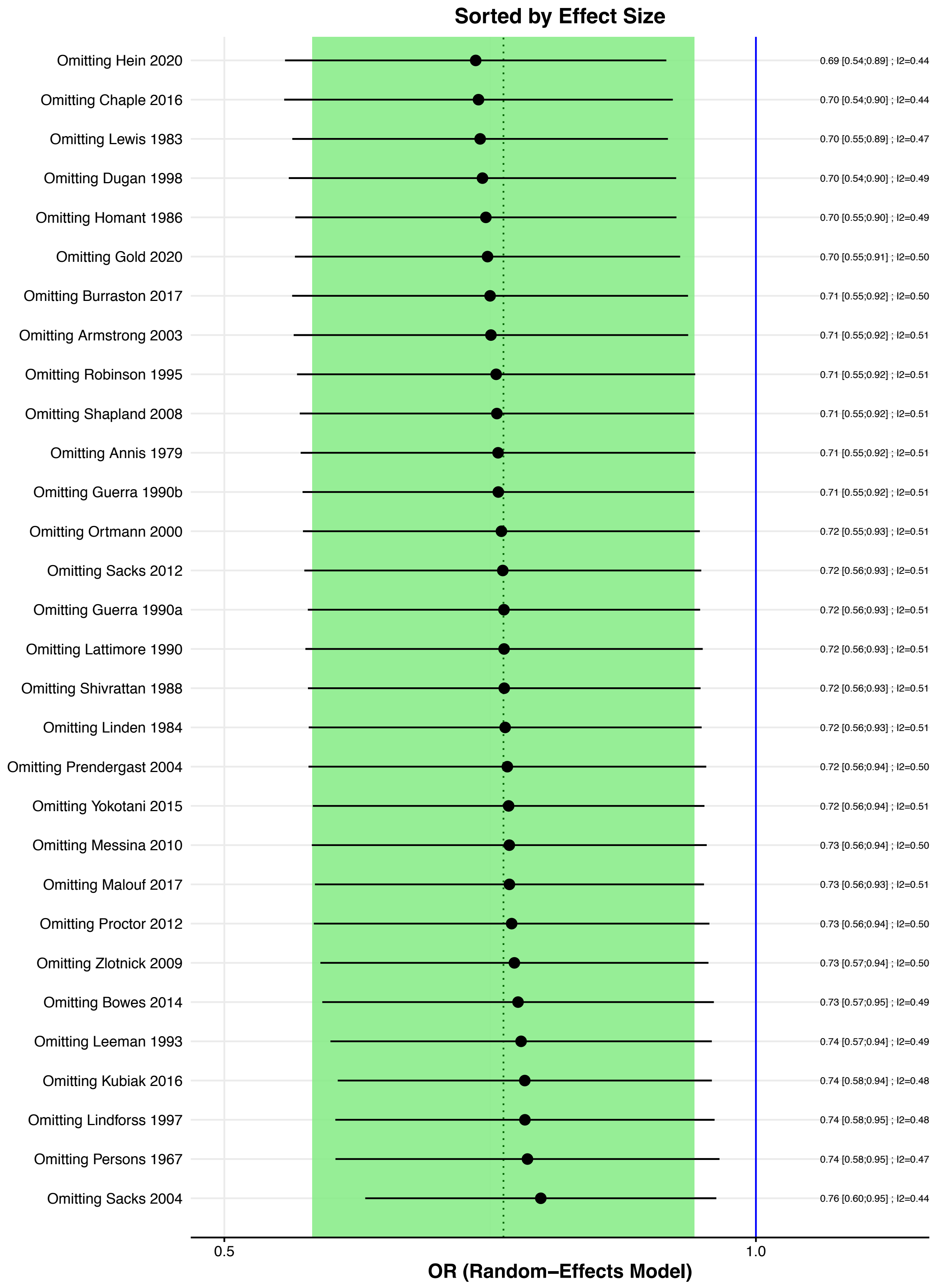

Figure A4. Baujat plot.

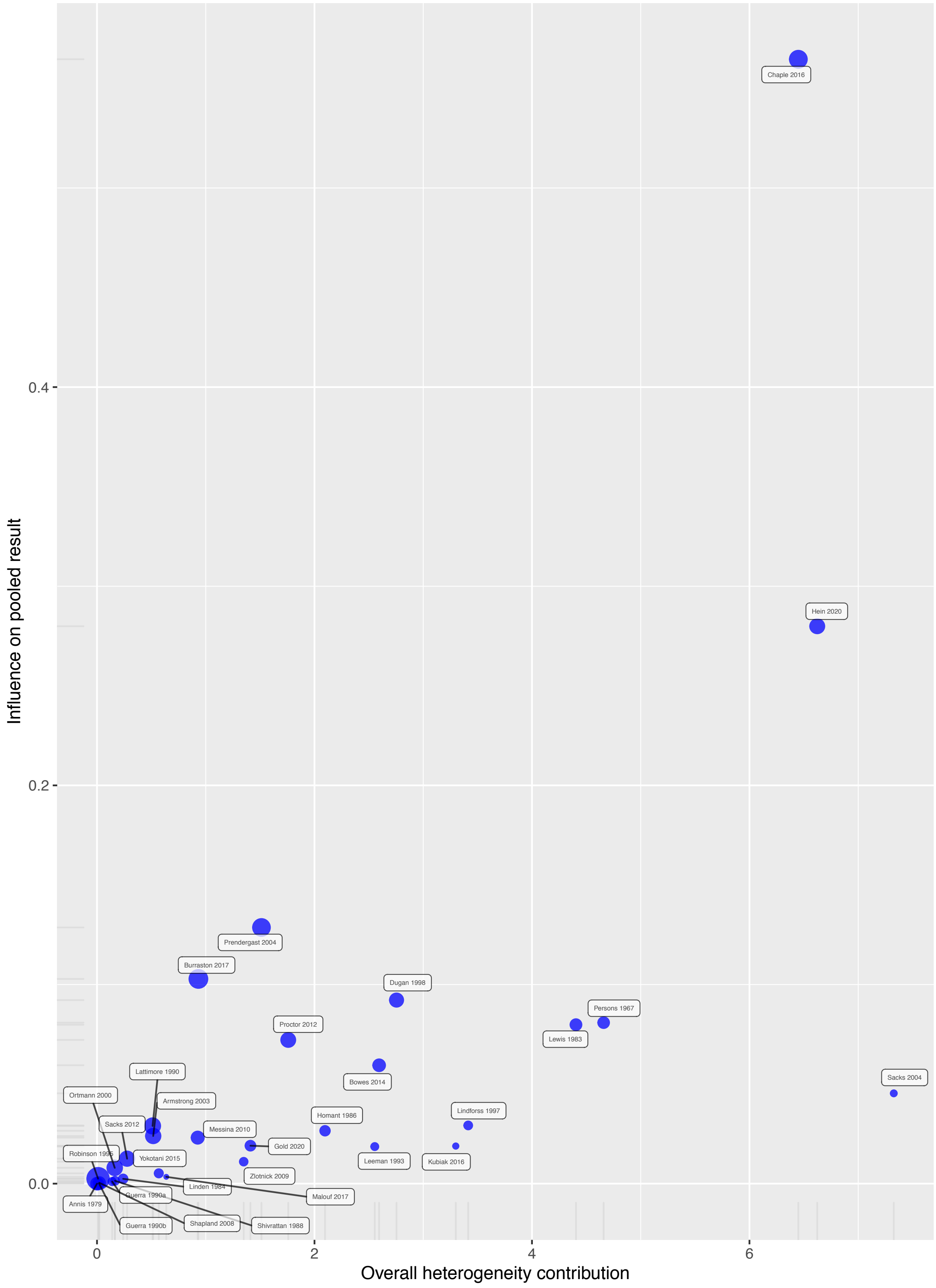

Figure A5. Funnel plot for all studies.

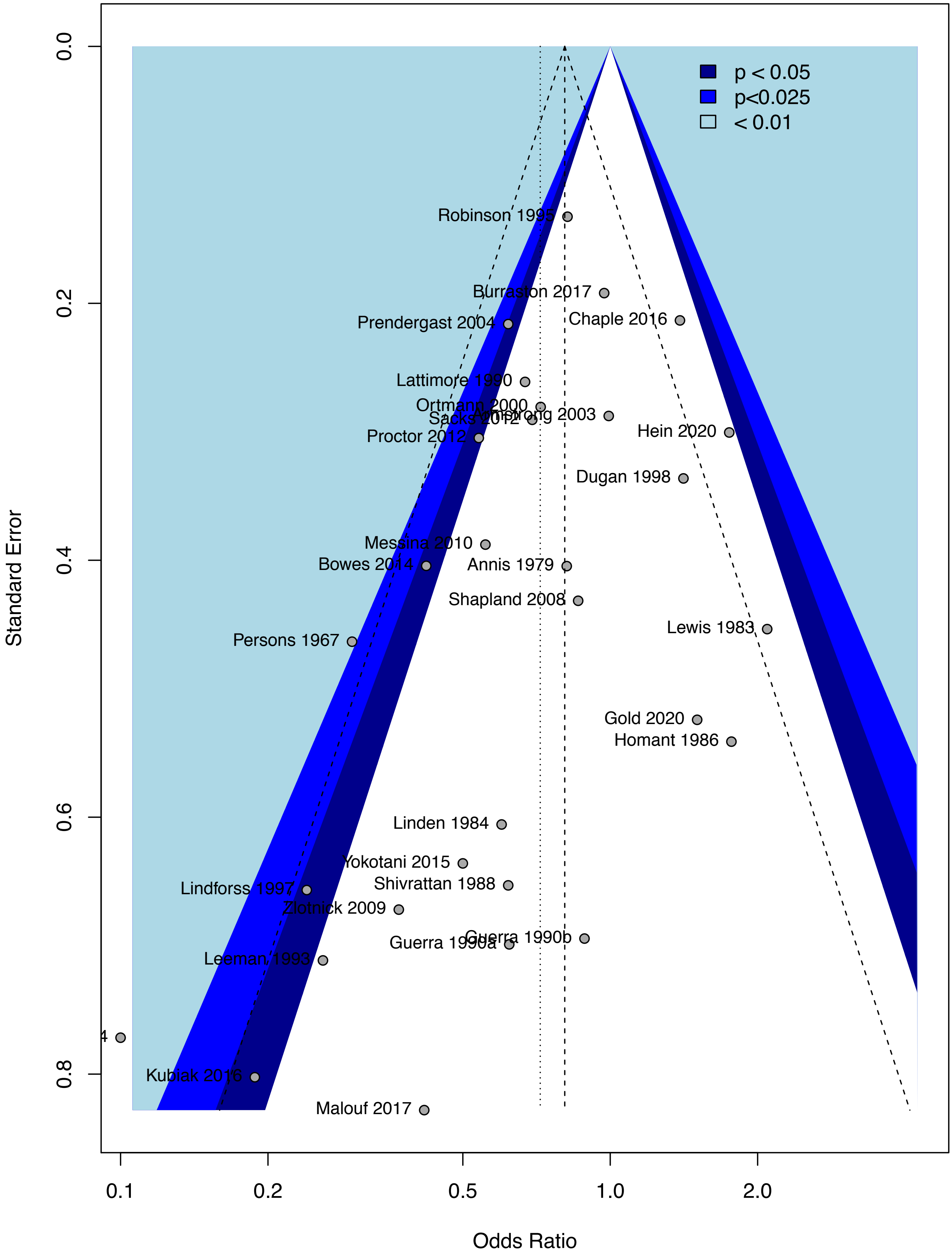

Figure A6. Funnel plot for all studies (including imputed studies).

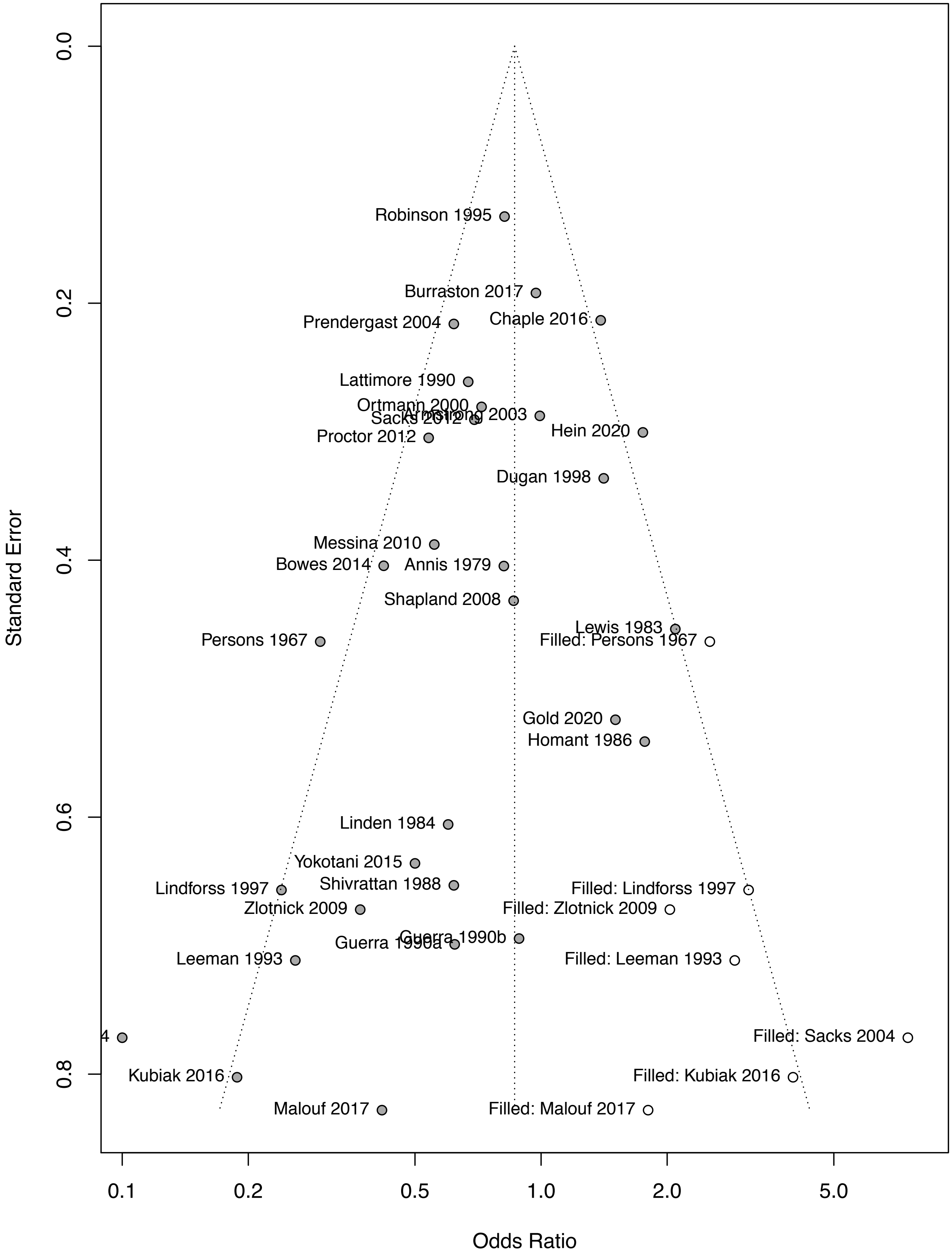

Figure A7. Forest plot of all studies (fixed-effect model).

Study	Intervention (n/N)	Control (n/N)	OR	95% CI	Weight
Persons 1967	13/41	25/41	0.30	[0.12; 0.74]	1.7%
Annis 1979	24/85	14/43	0.82	[0.37; 1.80]	2.2%
Lewis 1983	43/53	37/55	2.09	[0.86; 5.09]	1.8%
Linden 1984	20/30	20/26	0.60	[0.18; 1.97]	1.0%
Homant 1986	11/43	7/43	1.77	[0.61; 5.11]	1.3%
Shivrattan 1988	13/27	9/15	0.62	[0.17; 2.23]	0.9%
Lattimore 1990	50/138	50/109	0.67	[0.40; 1.12]	5.4%
Guerra 1990a	10/29	5.5/12	0.62	[0.16; 2.45]	0.7%
Guerra 1990b	12/28	5.5/12	0.89	[0.23; 3.46]	0.8%
Leeman 1993	3/20	15/37	0.26	[0.06; 1.04]	0.7%
Robinson 1995	371/1746	94/379	0.82	[0.63; 1.06]	20.8%
Lindforss 1997	18/30	25/29	0.24	[0.07; 0.87]	0.8%
Dugan 1998			1.41	[0.73; 2.73]	3.2%
Ortmann 2000	67/111	76/112	0.72	[0.42; 1.25]	4.6%
Armstrong 2003	71/110	66/102	0.99	[0.57; 1.75]	4.4%
Prendergast 2004	258/341	196/235	0.62	[0.41; 0.95]	7.8%
Sacks 2004	2/43	21/64	0.10	[0.02; 0.45]	0.6%
Shapland 2008	18/52	16/42	0.86	[0.37; 2.00]	2.0%
Zlotnick 2009	5/23	9/21	0.37	[0.10; 1.38]	0.8%
Messina 2010	19/60	25/55	0.56	[0.26; 1.19]	2.4%
Sacks 2012	27/207	29/163	0.69	[0.39; 1.23]	4.3%
Proctor 2012	50/98	56/85	0.54	[0.30; 0.98]	3.9%
Bowes 2014	27/52	41/57	0.42	[0.19; 0.93]	2.2%
Yokotani 2015	5/20	12/30	0.50	[0.14; 1.74]	0.9%
Kubiak 2016	3/19	8/16	0.19	[0.04; 0.91]	0.6%
Chaple 2016	67/244	51/238	1.39	[0.91; 2.11]	8.1%
Malouf 2017	10/16	12/15	0.42	[0.08; 2.11]	0.5%
Burraston 2017			0.97	[0.67; 1.42]	9.9%
Gold 2020	13/32	10/32	1.50	[0.54; 4.20]	1.3%
Hein 2020	98/118	126/171	1.75	[0.97; 3.15]	4.1%
**Fixed effect model**			**0.81**	**[0.72; 0.91]**	**100.0%**

Heterogeneity: $I^2 = 49\%$, $p < 0.01$

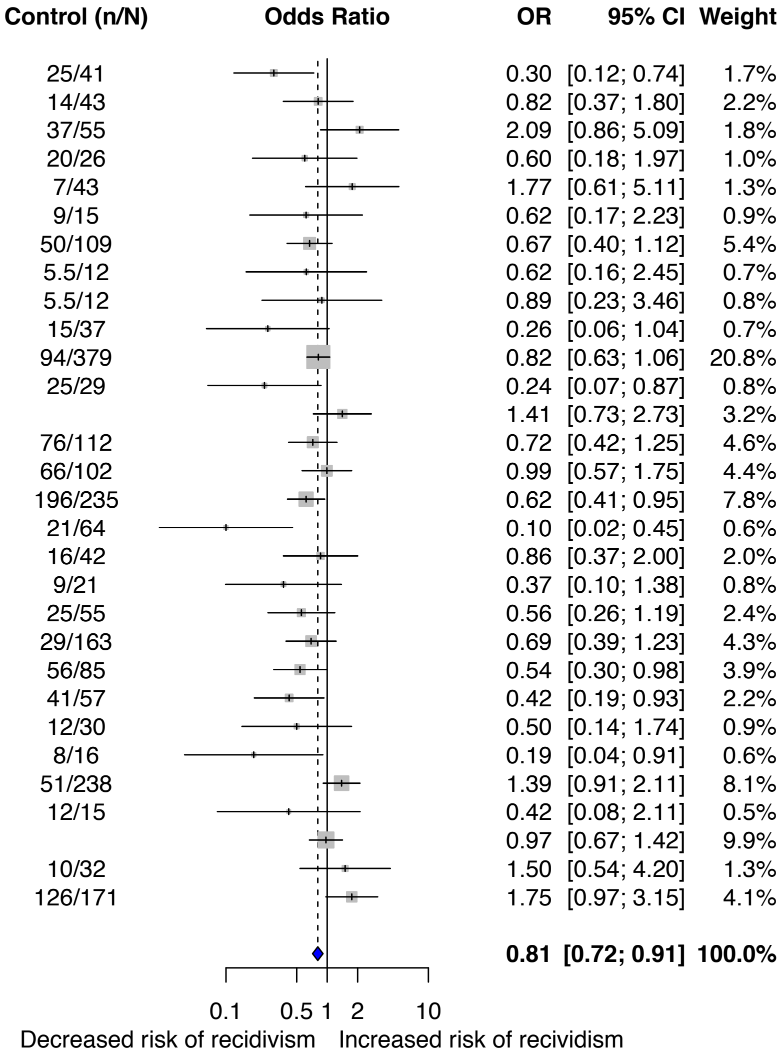

Note. Numbers of participants in the intervention and control groups are not available for Dugan 1998 and Burraston 2017, as the outcome was presented as continuous data rather than dichotomous data in both of these studies.

Figure A9. Forest plot of studies with an intervention group of ≥100 participants (random-effects model).

Study	Intervention (n/N)	Control (n/N)	OR	95% CI	Weight
Lattimore 1990	50/138	50/109	0.67	[0.40; 1.12]	10.2%
Robinson 1995	371/1746	94/379	0.82	[0.63; 1.06]	15.7%
Ortmann 2000	67/111	76/112	0.72	[0.42; 1.25]	9.5%
Armstrong 2003	71/110	66/102	0.99	[0.57; 1.75]	9.3%
Prendergast 2004	258/341	196/235	0.62	[0.41; 0.95]	12.0%
Sacks 2012	27/207	29/163	0.69	[0.39; 1.23]	9.2%
Chaple 2016	67/244	51/238	1.39	[0.91; 2.11]	12.1%
Burraston 2017			0.97	[0.67; 1.42]	13.0%
Hein 2020	98/118	126/171	1.75	[0.97; 3.15]	8.9%
**Random effects model**			**0.90**	**[0.71; 1.14]**	**100.0%**

Heterogeneity: $I^2 = 48\%$, $p = 0.05$

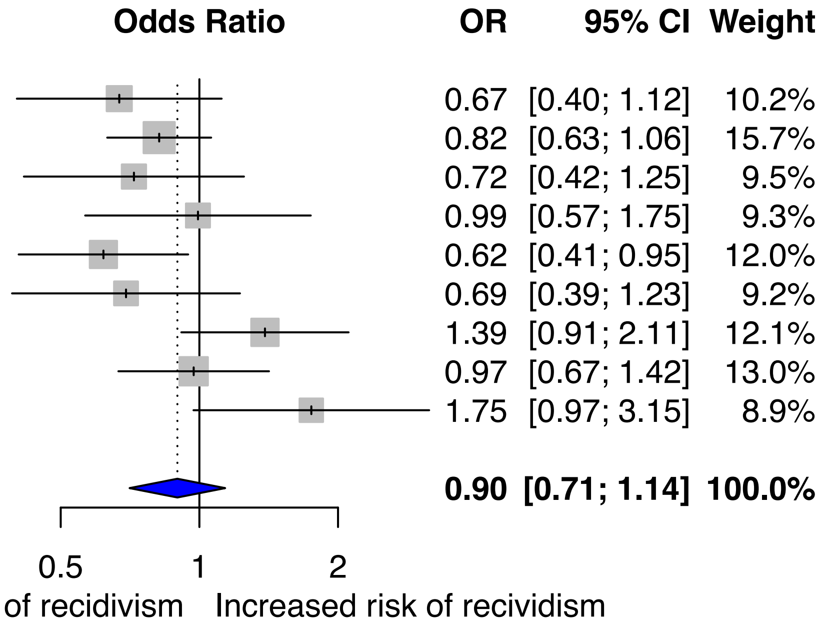

Note. Numbers of participants in the intervention and control groups are not available for Burraston 2017, as the outcome was presented as continuous data rather than dichotomous data in this study.
